# Real-world evidence study on the impact of SPECT MPI, PET MPI, cCTA and stress echocardiography on downstream healthcare utilisation in patients with coronary artery disease in the US

**DOI:** 10.1186/s12872-024-04225-y

**Published:** 2024-10-09

**Authors:** Matthieu Pelletier-Galarneau, Arturo Cabra, Erika Szabo, Santosh Angadageri

**Affiliations:** 1 Montreal Heart Institute, Montreal, QC Canada; 2 GE HealthCare, Marlborough, MA USA; 3 Clarivate Analytics, Toronto, Canada

**Keywords:** Coronary artery disease, Myocardial perfusion imaging, Downstream healthcare utilisation, Positron emission tomography, Single-photon emission computed tomography, Coronary computed tomography angiography, Stress echocardiography, Real-world evidence, Diagnostic accuracy, Coronary artery disease diagnosis

## Abstract

**Background:**

Coronary artery disease (CAD) is associated with a large clinical and economic burden. However, consensus on the optimal approach to CAD diagnosis is lacking. This study sought to compare downstream healthcare resource utilisation following different cardiac imaging modalities, to inform test selection for CAD diagnosis.

**Methods:**

Claims and electronic health records data from the Decision Resources Group Real-World Evidence US Data Repository were analysed for 2.5 million US patients who underwent single-photon emission computed tomography myocardial perfusion imaging (SPECT MPI), positron emission tomography myocardial perfusion imaging (PET MPI), coronary computed tomography angiography (cCTA), or stress echocardiography between January 2016 and March 2018. Patients were stratified into nine cohorts based on suspected or existing CAD diagnosis, pre-test risk, and prior events or interventions. Downstream healthcare utilisation, including additional diagnostic imaging, coronary angiography, and cardiac-related health system encounters, was compared by cohort and index imaging modality.

**Results:**

Among patients with suspected CAD diagnosed within 3 months of the index test, PET MPI was associated with lower downstream utilisation; 25–37% of patients who underwent PET MPI required additional downstream healthcare resources compared with 40–49% of patients who received SPECT MPI, 35–41% of patients who underwent cCTA, and 44–47% of patients who received stress echocardiography. Patients who underwent PET MPI experienced fewer acute cardiac events (5.3–9.4%) and generally had lower rates of healthcare encounters (0.8–4.1%) and invasive coronary angiography (ICA, 15.4–24.2%) than those who underwent other modalities. SPECT MPI was associated with more downstream ICA (31.3–38.2%) and a higher rate of cardiac events (9.5–13.2%) compared with PET MPI (5.3–9.4%) and cCTA (6.9–9.9%). Across all cohorts, additional diagnostic imaging was 1.6 to 4.7 times more frequent with cCTA compared with PET MPI.

**Conclusion:**

Choice of imaging modality for CAD diagnosis impacts downstream healthcare utilisation. PET MPI was associated with lower utilisation across multiple metrics compared with other imaging modalities studied.

**Supplementary Information:**

The online version contains supplementary material available at 10.1186/s12872-024-04225-y.

## Background

Coronary artery disease (CAD) occurs due to atherosclerosis in the coronary arteries and is a major cause of mortality and disability [1–4]. Individuals can be predisposed to CAD by a variety of genetic, environmental, and lifestyle risk factors [1]. Globally, CAD has a prevalence of 5–8% and accounts for approximately one-third of all deaths in individuals over the age of 35 years [3, 5]. In the US, CAD affects 16.8 million people and was associated with direct and indirect costs of $363 billion in 2016–2017 [6, 7]. Similarly, CAD accounts for annual costs of €169 billion in the European Union and £7.06 billion in the UK [8, 9]. CAD also exerts a substantial burden on low- and middle-income countries, where it is associated with 7 million deaths and 129 million disability adjusted life years annually, as well as monthly treatment costs of $300–1000 per patient [3, 10].

Traditionally, invasive coronary angiography (ICA) has been the gold standard for diagnosing CAD. However, due to its invasive nature, the technique is associated with risks [11, 12] as well as high costs [13, 14]. As a result, there has been a shift towards the use of non-invasive testing prior to the use of ICA [11]. Commonly used non-invasive tests include stress echocardiography, single-photon emission computed tomography (SPECT) myocardial perfusion imaging (MPI), positron emission tomography (PET) MPI, and coronary computed tomography angiography (cCTA) [11, 15–17]. Although a wealth of evidence on these techniques exists, there remains no consensus as to the optimal approach for evaluating stable ischaemic heart disease [18]. Instead, guidelines tend to focus on the value of different testing modalities in the context of clinical likelihood, patient presentations, and clinical profiles [17]. For example, in the AHA/ACC/ASE/CHEST/SAEM/SCCT/SCMR guidelines, cCTA is recommended for the evaluation of stable chest pain in intermediate/high risk patients < 65 years of age and not on optimal preventative therapies, while stress testing such as stress PET or SPECT MPI is preferred for those ≥ 65 years [19]. Conversely, National Institute for Health and Care Excellence (NICE) guidelines recommend cCTA when clinical assessment indicates typical or atypical angina or if abnormalities are found on a resting electrocardiogram (ECG), while non-invasive functional imaging is recommended if cCTA is non-diagnostic or shows CAD of uncertain functional significance [20]. However, it is also recognised that test selection can be influenced by other factors such as site expertise, availability, and cost [17, 19].

Non-invasive modalities can be classified into functional and anatomical approaches [11, 19]. Functional testing modalities, such as stress echocardiography, SPECT MPI and PET MPI, provide measures of ischaemia [11]. In contrast, cCTA provides anatomical data, enabling the assessment of coronary stenosis [11]. This distinction is of clinical importance as anatomical stenosis does not necessarily predict ischaemia or haemodynamic significance [17, 21, 22]. As such, the decision to use functional or anatomical modalities depends on the clinical question to be addressed.

There are also further distinctions between these techniques in their benefits and limitations. For example, SPECT MPI is a well-established technology that is used to a much greater extent than PET MPI, largely due to its broad availability, high diagnostic accuracy, and low cost [11, 23–25]. Furthermore, the introduction of new SPECT MPI technologies such as digital detectors, cardiac specific collimator systems, and ^18^F-labelled tracers have led to improved imaging quality and spatial resolution of MPI, enhanced diagnostic accuracy, more reliable quantification of perfusion, reduced radiation doses, and shorter MPI acquisitions [24, 26–29]. Stress echocardiography can also be used to visualise wall motion abnormalities. However, its accuracy is dependent on operator skill and can be compromised in patients with poor acoustic windows due to factors such as obesity or obstructive lung disease [30, 31]. On the other hand, a key advantage of cCTA is its ability to exclude diseases by differential diagnosis due to its excellent negative predictive value [32]. It is also particularly useful in the assessment of CAD in conditions of comprised immune system with increased prevalence of cardiovascular disease [33].

Importantly, the choice of imaging modality may influence downstream healthcare resource utilisation, including the use of additional imaging tests, ICA, therapeutic interventions, as well as resource use due to ischaemic events [23, 34]. For example, the ROMICAT-II study found that while the utilisation of cCTA in emergency departments improved clinical decision-making, it also increased the number of revascularisation procedures, downstream testing, and radiation exposure without any reduction in mortality or overall costs [35]. Similarly, our previous study of claims data and electronic healthcare records found that patients who underwent cCTA were more likely to undergo additional imaging tests compared with those who were tested with other imaging modalities [23].

To optimise the decision-making process in patients with (or suspected) CAD, evidence on the comparative benefits of imaging technologies is required. Such optimisation may reduce the need for subsequent ICA and other downstream resources, protecting patients from the risks associated with ICA and generating cost savings for healthcare systems [34]. Recent years have seen changes in usage and setting of cardiac imaging modalities [36, 37]; however, limited data is available on the impact of imaging modality choice on downstream healthcare utilisation. To address this, this real-world retrospective study of medical claims data in the US compared the impact of SPECT MPI, PET MPI, stress echocardiography, and cCTA on downstream healthcare utilisation, including additional diagnostic imaging, coronary angiography, and cardiac-related health system encounters and events.

## Methods

This was a real-world, retrospective analysis of medical claims data obtained from the Decision Resources Group (DRG) Real-World Evidence Data Repository US database. The database includes medical and pharmacy claims and electronic health records for > 300 million patients and covers approximately 98% of US health plans [38]. Source data are linked via a direct-matching algorithm and then de-identified at the patient level, permitting longitudinal tracking of patients across data sources and sites of care.

**Fig. 1:**
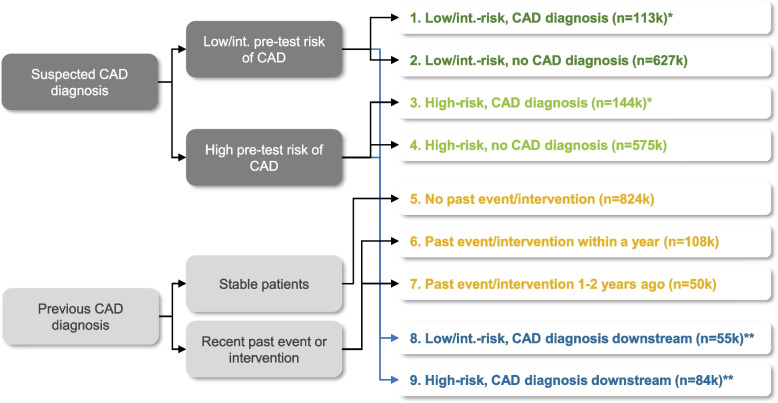
Stratification of patients. Note: *CAD diagnosis following index (reference) diagnostic test; **CAD diagnosis more than 3 months following index test. Abbreviations: CAD, coronary artery disease

The analysis included 2.5 million patients with CAD who underwent SPECT MPI, PET MPI, stress echocardiography, or cCTA between January 2016 and March 2018, as described previously [23]. For inclusion, patients were required to have at least two claims within a 1-year and 2-year period pre-index, and at least two claims within a 1-year period post-index. Patients were not included if the coefficient of variation (CV) for the number of claims (calculated as the sum of the standard deviation [SD] and mean for the sample, divided by the mean) was greater than the sum of the SD and mean for the sample. Using diagnostic codes in the claims data, patients were segmented into those with suspected CAD (Cohorts 1–4, 8 & 9) and those with an existing CAD diagnosis (Cohorts 5–7) at the index test (Fig. 1). Patients with suspected CAD were further segmented by the presence of a CAD diagnosis within 3 months (Cohorts 1–4) or more than 3 months following the index test (Cohorts 8 & 9).

Symptoms of chest pain are not captured in claims data; therefore, a proxy method was used to stratify low/intermediate-risk from high-risk patients based on sex, age, and underlying conditions (such as diabetes, hypertension, and hyperlipidaemia; see Figure S1, supporting information). Stratification of pre-test risk was informed by guidelines [19], simplified based on available data in the database. Patients with a previous CAD diagnosis were stratified based on the presence of acute cardiac events (acute coronary syndrome [ACS], unstable angina [UA], ST-elevation myocardial infarction [STEMI], non-STEMI [NSTEMI], ischaemic stroke [IS], transient ischaemic event [TIA], or acute heart failure [AHF]) and/or interventions (percutaneous coronary intervention [PCI] or coronary artery bypass graft [CABG] [23]. These groups were further stratified based on the recency of the past event/intervention (past event/intervention within 1 year pre-index or within 1–2 years pre-index).

### Statistical analyses

Patients were stratified into nine cohorts (Fig. 1). The number of patients in each segment was identified and matched based on age, sex, heart failure and atrial fibrillation/flutter/other cardiac arrhythmias. Patient groups were comparable in age, sex, and other variables, with a 1:1 male to female ratio, supporting a fair comparison. Because many group comparison tests were performed, statistical variance can vary. A p-value cut-off of 0.05 was therefore not considered sufficient, and so a more stringent cut-off of 0.005 was used in this analysis.

Downstream utilisation was defined in this study as the use of diagnostic tests (additional diagnostic imaging or ICA after the index test), occurrence of events (ACS events, including UA, STEMI, NSTEMI, IS, TIA, AHF), use of interventions (PCI, CABG), and occurrence of cardiac-related encounters (hospitalisations, cardiac office, emergency room [ER]). Linear and logistic regression was used to analyse numeric downstream variables (e.g. count of events, count of interventions) and categorical downstream variables (e.g. presence of events, presence of interventions) on the effects of SPECT MPI, PET MPI and cCTA on downstream healthcare utilisation.

## Results

### Aggregate analysis of downstream healthcare utilisation

We have reported characteristics of each cohort in previous work [23]. Our preliminary analysis found that of patients who received a standalone imaging test, 77% of patients received SPECT MPI, 18% underwent stress echocardiography, 3% received PET MPI, and 2% received cCTA [23]. A further analysis of physician referral patterns found that 43% of physicians referred almost all (> 90%) of their patients to SPECT MPI, while just 3%, 1% and 1% of physicians referred > 90% of their patients to stress echocardiography, PET MPI or cCTA, respectively [23].

In this study, an analysis of post-imaging downstream healthcare utilisation for all patients in the aggregate reveals broadly comparable downstream healthcare utilisation for SPECT MPI, PET MPI, and cCTA, while that for stress echocardiography was generally lower (Table 1).

**Table 1:** Post-imaging downstream healthcare utilisation (all patients)

	**Additional diagnostic tests**	**Coronary angiography**	**Acute coronary syndrome event**	**Other events** ^**a**^	**ER encounters**	**Emergent interventions** ^**b**^	**Planned intervention** ^**b**^	**Inpatient encounters**
**Stress echocardiography**	2.9%	6.0%	2.0%	1.2%	1.0%	0.2%	1.6%	1.8%
**SPECT MPI**	3.4%	12.4%	4.4%	3.3%	2.0%	0.5%	3.4%	3.5%
**PET MPI**	2.2%	12.8%	4.6%	4.1%	1.9%	0.6%	5.0%	3.6%
**cCTA**	6.6%	10.9%	4.3%	3.2%	2.1%	0.4%	3.7%	8.0%

*Abbreviations* *:* *cCTA* Coronary computed tomography angiography, *ER* Emergency room, *PET MPI* Positron emission tomography myocardial perfusion imaging, *SPECT MPI* Single-photon emission computed tomography myocardial perfusion imaging

^a^Other events’ includes ischaemic stroke, transient ischaemic event, and acute heart failure

^b^Emergent’ and ‘Planned’ interventions denote the presence or absence, respectively, of myocardial infarction on the claim

Use of cCTA at the index test was associated with the highest rate of additional downstream imaging (6.6%), while PET MPI resulted in the lowest (2.2%). A similar proportion of patients diagnosed by PET MPI (12.8%) and SPECT MPI (12.4%) received downstream ICA; this was marginally higher than for patients who underwent cCTA (10.9%), and approximately double those who underwent stress echocardiography (6.0%). Compared with stress echocardiography (1.8%), SPECT MPI (3.5%), and PET MPI (3.6%), a larger proportion of patients who underwent cCTA (8.0%) at the index test were subsequently admitted to hospital, while patients who underwent PET MPI (5.0%) were more likely to have planned interventions compared with those who underwent stress echocardiography (1.6%), SPECT MPI (3.4%) or cCTA (3.7%). A similar proportion of patients were admitted to an emergency room (stress echocardiography, 1.0%; SPECT MPI, 2.0%; PET MPI, 1.9%; cCTA, 2.1%) or received interventions associated with myocardial infarctions (stress echocardiography, 0.2%; SPECT MPI, 0.5%; PET MPI, 0.6%; cCTA, 0.4%), regardless of index test imaging modality.

### Downstream healthcare utilisation by cohort

**Fig. 2:**
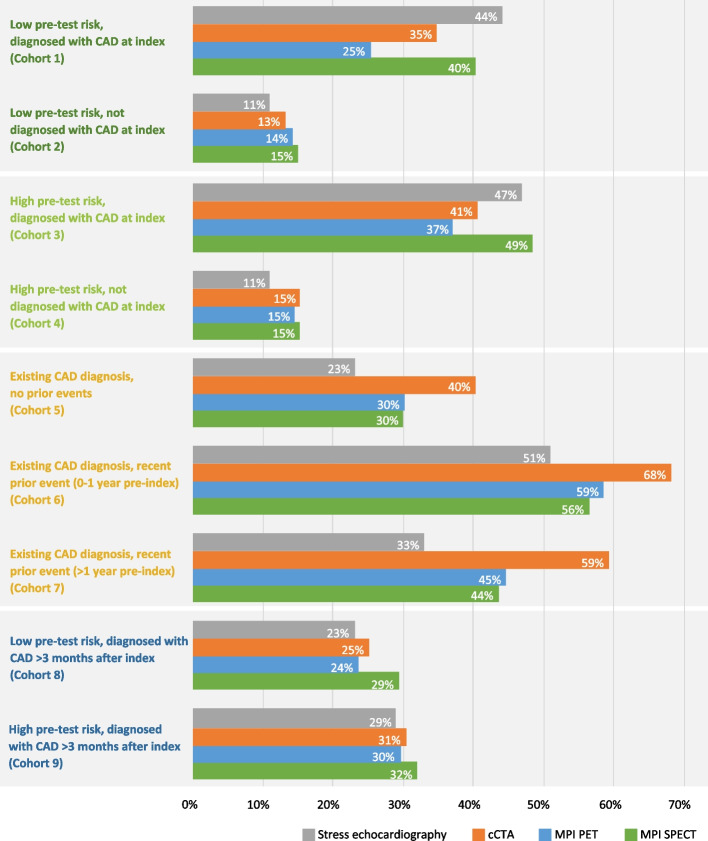
Proportion of patients utilising any healthcare resource^†^ downstream. ^†^Healthcare resources included additional diagnostic imaging, coronary angiography, interventions (PCI, CABG), hospital inpatient or outpatient visits, cardiac office visits, and emergency visits. Abbreviations: CABG, coronary artery bypass graft; CAD, coronary artery disease; cCTA, coronary computed tomography angiography; PCI, percutaneous coronary intervention; PET MPI, positron emission tomography myocardial perfusion imaging; SPECT MPI, single-photon emission computed tomography myocardial perfusion imaging

While an analysis of downstream healthcare utilisation in the aggregate is useful for understanding broad trends, it does not reveal valuable information on clinically differentiated patient populations. To determine the impact of various imaging tests on downstream healthcare utilisation for specific patient segments, a more detailed examination of these data by cohort was conducted. This approach showed clear differences in downstream utilisation by imaging modality between the cohorts (Fig. 2). A breakdown of downstream utilisation for each cohort, including the proportion of patients receiving additional diagnostic imaging, ICA, interventions, as well as those experiencing cardiac-related health system encounters and events is presented in Table S1 and Figures S2–6 (supporting information). An additional comparison of all recorded downstream occurrences for patients who initially underwent SPECT MPI or PET MPI is provided in Table 2.

**Table 2:** Comparisons of downstream occurrences between SPECT MPI and PET MPI for each cohort

**Type of occurrence**	**Occurrence of events**	**Cohort 1, Low risk, CAD Dx**		**Cohort 2, Low risk, no CAD Dx**		**Cohort 3, High risk, CAD Dx**		**Cohort 4, High risk, no CAD Dx**		**Cohort 5, CAD, no prior events**		**Cohort 6, CAD, event past 1 year**		**Cohort 7, CAD, event 1–2 years ago**	
		**SPECT**	**PET**	**SPECT**	**PET**	**SPECT**	**PET**	**SPECT**	**PET**	**SPECT**	**PET**	**SPECT**	**PET**	**SPECT**	**PET**
**Events**	**Any events**	9%	**5%**	2%	2%	13%	**9%**	3%	3%	8%	8%	**32%**	37%	**17%**	18%
	**ACS**	8%	**4%**	1%	**0%**	10%	**6%**	1%	1%	5%	5%	**19%**	20%	**10%**	11%
	**Non-ACS**	2%	2%	1%	1%	4%	4%	**2%**	3%	3%	3%	**16%**	22%	**8%**	9%
	**IS or TIA**	1%	1%	1%	1%	2%	2%	1%	1%	2%	**1%**	6%	**5%**	4%	**3%**
	**UA**	6%	**3%**	0%	0%	8%	**4%**	0%	0%	4%	4%	11%	11%	**7%**	8%
	**STEMI**	1%	**0%**	0%	0%	1%	1%	0%	0%	1%	**0%**	3%	3%	1%	1%
	**NSTEMI**	2%	**1%**	0%	0%	2%	**1%**	0%	0%	1%	1%	**8%**	9%	3%	3%
	**IS**	1%	1%	1%	1%	2%	2%	1%	1%	1%	1%	5%	5%	3%	3%
	**TIA**	0%	0%	0%	0%	0%	0%	0%	0%	0%	0%	**0%**	1%	0%	0%
	**AHF**	1%	1%	**0%**	1%	2%	2%	1%	1%	2%	2%	**11%**	18%	**4%**	6%
**Additional diagnostic tests or procedures**	**CMRA**	0%	0%	0%	0%	0%	0%	0%	0%	0%	0%	0%	0%	0%	0%
	**Stress echo**	0%	0%	0%	0%	0%	0%	0%	0%	0%	0%	1%	1%	0%	0%
	**MPI SPECT**	3%	**1%**	2%	**0%**	4%	**1%**	2%	**1%**	3%	**1%**	7%	**3%**	5%	**2%**
	**MPI PET**	0%	0%	0%	0%	**0%**	1%	**0%**	1%	**0%**	1%	**0%**	3%	**0%**	1%
	**cCTA**	**0%**	1%	0%	0%	**0%**	1%	0%	0%	0%	0%	0%	0%	0%	0%
	**Coronary angiography **	31%	**15%**	3%	**2%**	38%	**24%**	3%	3%	15%	15%	**24%**	27%	20%	20%
**Interventions and health system encounters**	**PCI**	8%	**5%**	0%	0%	10%	**8%**	0%	0%	**5%**	6%	**10%**	14%	8%	8%
	**CABG**	1%	1%	0%	0%	2%	**1%**	0%	0%	0%	0%	**1%**	2%	1%	1%
	**Cardiac inpatient claims **	3%	**2%**	1%	1%	6%	**4%**	2%	2%	4%	**3%**	**12%**	14%	8%	8%
	**Cardiac outpatient claims **	4%	**3%**	2%	2%	5%	**4%**	**2%**	3%	4%	4%	**9%**	11%	**6%**	8%
	**Cardiac office claims **	6%	6%	**4%**	6%	**6%**	7%	**4%**	5%	**8%**	11%	**9%**	12%	**8%**	15%
	**Cardiac ER claims **	2%	**1%**	1%	1%	3%	**2%**	1%	1%	2%	2%	8%	8%	5%	**4%**
	**Cardiac office claims and cardiac outpatient claims **	9%	**8%**	**6%**	8%	10%	10%	**5%**	7%	**11%**	14%	**16%**	21%	**13%**	21%
	**FFR**	0%	0%	0%	0%	0%	0%	0%	0%	0%	0%	0%	0%	0%	0%
	**Any imaging**	4%	**2%**	2%	**1%**	5%	**3%**	3%	**2%**	3%	**2%**	8%	**6%**	6%	**3%**
	**Interventions **	9%	**6%**	0%	0%	12%	**10%**	0%	0%	**5%**	7%	**11%**	16%	**8%**	9%
	**Interventions with MI **	1%	**0%**	0%	0%	1%	1%	0%	0%	1%	1%	**3%**	4%	1%	1%
	**Interventions without MI **	8%	**5%**	0%	0%	11%	**9%**	0%	0%	**5%**	6%	**9%**	13%	**7%**	8%

For each MPI SPECT/MPI PET comparison, the lowest value is shown in bold

*Abbreviations: ACS* Acute coronary syndrome, *AHF* Acute heart failure, *CABG* Coronary artery bypass graft, *CAD* Coronary artery disease, *cCTA* Coronary computed tomography angiography, *CMRA* Cardiac magnetic resonance angiography, *Dx* Diagnosis, *ER* Emergency room, *FFR* Fractional flow reserve, *IS* Ischaemic stroke, *MI* Myocardial infarction, *MPI* Myocardial perfusion imaging, *NSTEMI* Non-ST-elevation myocardial infarction, *PCI* Percutaneous coronary intervention, *PET* Positron emission tomography, *SPECT* Single-photon emission computed tomography, *STEMI* ST-elevation myocardial infarction, *TIA* Transient ischaemic event, *UA* Unstable angina

Across all cohorts, rates of additional non-invasive diagnostic imaging were highest for patients who initially underwent cCTA (3.6–12.4%) and lowest for those who underwent PET MPI (1.3–6.2%) (Table S1, supporting information). A comparison of downstream imaging rates between the four modalities is provided in Table 3. Ratios of downstream imaging rates between patients who underwent PET MPI and those who received cCTA ranged from 1.6-fold higher rates for cCTA in cohort 6 to 4.7-fold higher in Cohort 1 (Table 3).

**Table 3:** Comparisons of rates of any downstream imaging between different initial testing modalities for each cohort

**Cohort**	**Any downstream imaging**			
	**PET MPI**	**cCTA**	**SPECT MPI**	**SE**
**Low pre-test risk, diagnosed with CAD at index (Cohort 1)**	**1.7%**	8.0%	3.5%	4.2%
**Low pre-test risk, not diagnosed with CAD at index (Cohort 2)**	**1.3%**	3.6%	2.1%	1.8%
**High pre-test risk, diagnosed with CAD at index (Cohort 3)**	**2.8%**	10.1%	4.7%	5.0%
**High pre-test risk, not diagnosed with CAD at index (Cohort 4)**	**1.7%**	4.4%	2.6%	2.4%
**Existing CAD diagnosis, no prior events (Cohort 5)**	**1.9%**	7.3%	3.5%	3.8%
**Existing CAD diagnosis, recent prior event (0–1 year pre-index) (Cohort 6)**	**6.2%**	10.3%	7.6%	8.8%
**Existing CAD diagnosis, recent prior event (>1 year pre-index) (Cohort 7)**	**3.1%**	12.4%	5.7%	5.8%
**Low pre-test risk, diagnosed with CAD >3 months after index (Cohort 8)**	**2.7%**	6.7%	4.8%	6.1%
**High pre-test risk, diagnosed with CAD >3 months after index (Cohort 9)**	**4.0%**	9.1%	6.0%	7.1%

For each comparison, the lowest value is shown in bold

*Abbreviations: cCTA* Coronary computed tomography angiography, *PET MPI* Positron emission tomography myocardial perfusion imaging, *SE* Stress echocardiography, *SPECT MPI* Single-photon emission computed tomography myocardial perfusion imaging

Among patients with suspected CAD (low or high risk) who were diagnosed within 3 months of their index test (Cohorts 1 and 3), the use of PET MPI as the modality of initial diagnosis resulted in lower use of additional healthcare resources compared with other modalities (Fig. 2 and Table S1, supporting information). This difference was most apparent for low-risk patients (Cohort 1), with 25% of patients who underwent PET MPI utilising any downstream healthcare, compared with 44%, 40% and 35% of patients who underwent stress echocardiography, SPECT MPI or cCTA. A similar trend was observed for high-risk patients (Cohort 3), although the differences were less pronounced (stress echocardiography, 47%; PET MPI, 37%; SPECT MPI, 49%; cCTA 41%).

Among patients with suspected CAD (low or high risk) who did not receive a diagnosis following the index test, the proportion of patients utilising downstream healthcare for stress echocardiography, SPECT MPI, PET MPI, and cCTA was comparable for both low-risk (Cohort 2) and high-risk (Cohort 4) patients.

For patients with an existing CAD diagnosis at the time of the index test (Cohorts 5–7), the likelihood of downstream utilisation was greater with cCTA compared with stress echocardiography, PET MPI and SPECT MPI (Fig. 2). This difference in downstream utilisation was mostly due to additional diagnostic imaging and cardiac-related encounters, with additional diagnostic imaging 1.6 to 4.0 times more frequent with cCTA compared with PET MPI (Table 3; Table S1, supporting information). Patients with CAD and prior cardiac events or interventions (Cohorts 6 and 7) generally reported higher rates of downstream healthcare utilisation (33–68%) than other cohorts studied, with particularly high rates of cardiovascular events and cardiac-related health system encounters (Table S1, supporting information). As reported previously, patients in these cohorts tended to have more severe and symptomatic disease, in addition to a larger comorbidity burden [23].

In patients who underwent combinations of imaging tests, downstream utilisation was lowest in those who received SPECT MPI followed by PET MPI compared with all other combinations examined (SPECT MPI followed by cCTA or stress echocardiography followed by either cCTA or SPECT MPI) in all cohorts other than 6 (Figure S 7, supporting information).

### Downstream interventions, cardiac-related encounters, and events by cohort

In patients with suspected CAD (low or high risk) who were diagnosed within 3 months of the index test (Cohorts 1 and 3), patients diagnosed with SPECT MPI were more likely to undergo coronary angiography (31.3–38.2%) compared with those diagnosed by PET MPI (15.4–24.2%), cCTA (17.4–21.5%), or stress echocardiography (31.0–36.5%) (Table 2; Table S1, supporting information).

Those who underwent PET MPI or cCTA were also less likely to have downstream interventions (PCI or CABG) than SPECT MPI or stress echocardiography (Table S1, supporting information). In low pre-test risk patients (Cohort 1), a higher proportion of patients who underwent stress echocardiography (10.1%) and SPECT MPI (9.0%) utilised downstream interventions, compared with patients diagnosed by PET MPI (5.8%) or cCTA (6.4%). Similarly, for high-risk patients (Cohort 3), a marginally higher proportion of patients diagnosed by stress echocardiography (13.1%) and SPECT MPI (11.9%) at the index test received PCI or CABG than those diagnosed by PET MPI (9.6%) or cCTA (8.4%) (Table 2; Table S1, supporting information).

Both low- and high-risk patients with suspected CAD who were subsequently diagnosed within 3 months (Cohorts 1 and 3) of a PET MPI test typically experienced lower rates of hospital inpatient, outpatient and emergency department encounters (0.8–4.1%) compared with those who underwent SPECT MPI (1.5–5.7%), cCTA (1.2–7.5%) or stress echocardiography (1.1–6.6%) (Table 2; Table S1, supporting information). Higher rates of cardiac office visits were reported for patients diagnosed by PET MPI (6.2–7.2%) compared with SPECT MPI (5.6–5.8%), cCTA (4.2–4.7%), or stress echocardiography (4.7%). As expected, patients not diagnosed with CAD following the index test (Cohorts 2 and 4) experienced lower rates of cardiac-related health system encounters (0.4–6.1%) than those who received a CAD diagnosis (0.8–7.5%) (Cohorts 1 and 3).

Among patients with suspected CAD who were diagnosed following the index test (Cohorts 1 and 3), a smaller proportion who underwent PET MPI experienced downstream cardiac events (low pre-test risk, 5.3%; high pre-test risk, 9.4%), compared with those diagnosed by SPECT MPI (low pre-test risk, 9.5%; high pre-test risk, 13.2%), cCTA (low pre-test risk, 6.9%; high pre-test risk, 9.9%), or stress echocardiography (low pre-test risk, 8.6%; high pre-test risk, 12.3%) (Table 2; Table S1, supporting information). ACS was the most common acute cardiac event reported by patients in Cohorts 1 and 3, regardless of index testing modality. Across all cohorts and index imaging modalities, ACS events were more commonly encountered in patients who received additional testing (Figures S 2–6, supporting information).

## Discussion

This large, real-world, retrospective study of US claims data evaluated downstream healthcare utilisation in patients who underwent a diagnostic imaging test for CAD. Among patients with suspected CAD who were diagnosed within 3 months of their index test, use of PET MPI was associated with lower downstream healthcare utilisation than SPECT MPI, cCTA, or stress echocardiography, across multiple metrics. This trend was true for both low (Cohort 1) and high (Cohort 3) pre-test risk patients but was more pronounced for the low-risk patient cohort. For patients who did not receive a CAD diagnosis following their index test (Cohorts 2 and 4), comparable downstream healthcare utilisation was observed between the four imaging modalities. The higher downstream healthcare utilisation in patients diagnosed with CAD at the index test (Cohorts 1 and 3) compared with those without CAD at the index test (Cohorts 2 and 4) is expected and reflects the fact that these patients have the disease and require downstream tests and procedures. Among patients with CAD who had previously experienced cardiac events or interventions (Cohorts 6 and 7), those who underwent more advanced imaging tests such as PET MPI or cCTA tended to have higher downstream utilisation compared with those who underwent SPECT MPI or stress echocardiography. However, the interpretation of data for these cohorts is difficult due to their more complex epidemiological profiles [23]. As previously reported, patients who underwent different types of imaging tests in these cohorts also differed in factors such as prior events and interventions [23]. For example, those who underwent PET MPI or cCTA were more likely to have experienced acute heart failure [23].

Patients with suspected CAD who received a diagnosis within 3 months of their PET MPI test subsequently experienced fewer acute cardiac events, and typically had lower rates of hospital inpatient, outpatient, and emergency encounters compared with those who underwent stress echocardiography, cCTA, or SPECT MPI. These patients did, however, experience higher rates of cardiac office visits. This finding suggests that patients who are diagnosed with CAD following PET MPI are more likely to be referred to cardiology specialists, with a resulting improvement in acute cardiac events compared with other modalities. Interestingly, in patients with suspected CAD who received a diagnosis within 3 months, SPECT MPI was associated with more ICA but also higher rates of events, suggesting that utilisation of SPECT MPI leads to more unnecessary ICA. This finding can inform guidelines on the use of non-invasive diagnostic tests for CAD, particularly regarding the potential benefits of a greater emphasis on PET MPI. As expected, patients without a diagnosis of CAD experienced lower rates of downstream acute cardiac events than those with a diagnosis.

Across all cohorts and index imaging modalities, ACS events were more commonly encountered in patients who received additional testing. Many patients who receive additional diagnostic tests (most commonly coronary angiography) experienced a subsequent event, while those who did not receive additional testing experienced very low incidence of events. This likely reflects a tendency for patients with more severe CAD to receive additional testing.

We have previously reported rates of downstream imaging and ICA in each cohort [23]. The similar use of additional downstream imaging observed for cCTA and PET MPI in patients with suspected CAD who did not receive a diagnosis of CAD within 3 months (Cohorts 2 and 4) may be attributable to the low false negative rate associated with cCTA. Although cCTA is regarded as a reliable technique for ruling out a CAD diagnosis [30], the positive predictive value of the technique is inferior to that of PET MPI, making cCTA a less accurate option for identifying obstructive CAD [39]. Similarly, SPECT MPI can generate false positive results in the presence of attenuation artefacts. Together, these factors may contribute to the higher rates of downstream healthcare utilisation among patients who underwent cCTA and SPECT MPI at the index test compared with PET MPI in Cohorts 1 and 3.

Among patients who received combinations of tests, the SPECT MPI/PET MPI combination had the lowest downstream utilisation compared with stress echocardiography/SPECT MPI or stress echocardiography/cCTA or SPECT MPI/cCTA for both patients with suspected and existing CAD diagnosis at index test. However, it should be noted that this analysis did not include patients who first underwent PET MPI. Lower confidence among referring physicians in the validity of positive results with stress echocardiography or SPECT MPI may lead to the use of further confirmatory tests. PET MPI has a superior sensitivity and diagnostic accuracy for CAD diagnosis compared with SPECT MPI [24, 40], especially in people with obesity or women with dense breast tissue, making it a more appropriate initial test or one that may be needed after other tests in these populations [41].

Choice of diagnostic imaging modality among patients with suspected CAD was also found to affect the likelihood of downstream ICA [23]. The lower rates of ICA following PET MPI compared with SPECT MPI suggests cardiac imaging tests provide value in their ability to efficiently guide post-imaging patient management. Understanding and optimising imaging pathways in patients with suspected CAD may reduce the need for wasted resources due to unnecessary downstream ICA or additional imaging. These findings highlight the potential role of first-line advanced non-invasive imaging in optimising healthcare resource utilisation through informed decision on test suitability for different patient populations.

**Fig. 3:**
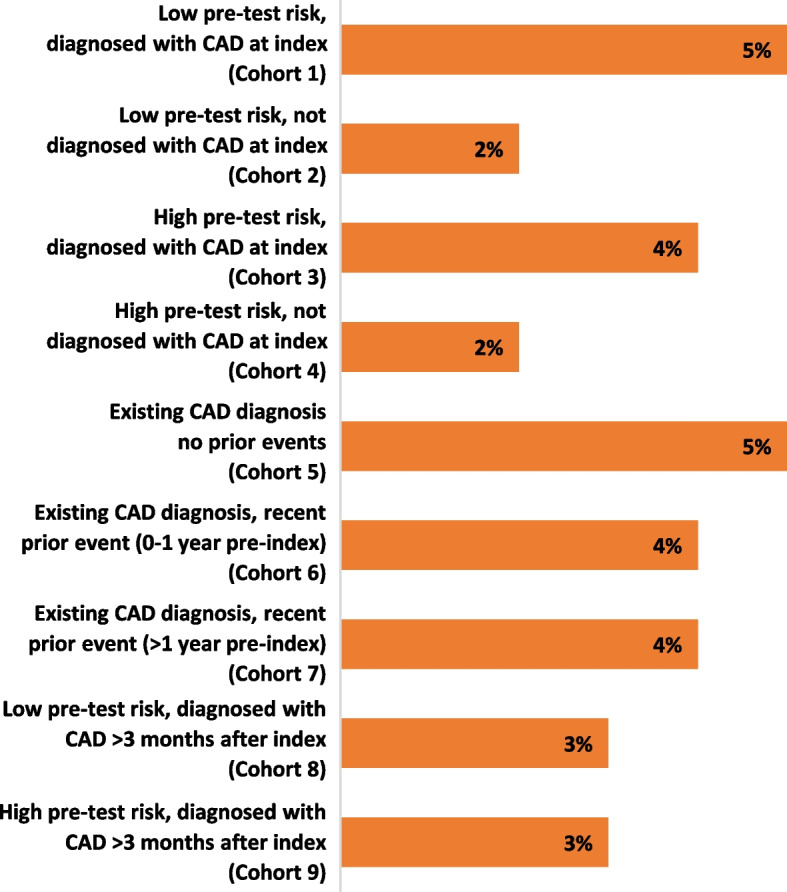
Percentage of patients in each cohort receiving standalone PET MPI. Abbreviations: CAD, coronary artery disease; PET MPI, positron emission tomography myocardial perfusion imaging

PET MPI offers several technical advantages compared with SPECT MPI, including higher spatial resolution, superior attenuation correction, and accurate myocardial blood flow quantification, which result in improved diagnostic accuracy [24, 40, 42, 43]. However, despite the diagnostic advantages of PET MPI and its apparent benefits in terms of reduced downstream healthcare utilisation, the technique is still not widely used [17, 41, 42, 44, 45]. As reported previously, PET MPI did not exceed 5% of total standalone imaging in any cohort (Fig. 3) [23]. In contrast, the use of standalone MPI SPECT in Cohorts 1–9 ranged from 66 to 86% [23]. The broader use of SPECT MPI compared with PET MPI therefore likely reflects the greater accessibility of this technique, as well as factors such as high cost of training and equipment for PET MPI compared with other modalities [11, 23, 40, 46, 47]. A comparison of imaging modalities reported that the cost per test for PET MPI is approximately $1,800, compared with $1,600 for both SPECT MPI and cCTA [11]. However, differences in costs may be negligible if reimbursed by insurance in countries such as the US. The use of PET MPI may lead to less downstream healthcare resource utilisation compared with other modalities due to improved diagnostic accuracy [24, 42, 43]. The associated savings may be realised in disease management by cardiologists, less invasive interventional procedures, fewer hospital admissions, and fewer unnecessary repetitions of diagnostic tests. Communicating these benefits of PET MPI to referring physicians and other decision-makers may help increase usage of this imaging technique to optimise patient management.

As with other secondary analyses, the analysis of medical claims data in this study is associated with several limitations. As claims data are often adjudicated on an ongoing basis, healthcare services provided by out-of-network providers may not be captured by the database. Real-world data obtained from routine practice may also be prone to missing and erroneous data, imperfect codes, a lack of standardisation of clinical measures, differences between clinical testing centres, varying measurement periodicity, and inconsistencies in recording certain covariates of interest [48]. Additionally, while patients were stratified based on sex, age and underlying condition (i.e. diabetes, hypertension, hyperlipidaemia and smoking), the study did not account for family history of CAD and previous coronary artery calcium testing, which may impact risk assessment and thus choice of imaging modality [19]. Finally, this study was conducted in a US population, and may not be generalisable to other regions. In England, for example, following the 2016 publication of the NICE Clinical Guideline Number 95 (CG95), which recommended cCTA as first-line test for possible angina, studies indicate increased use of cCTA and modest reductions in the use of ICA [37]. This has been associated with fewer hospitalisations for myocardial infarction and a decline in CAD mortality [37]. However, the data presented herein provide useful insights on healthcare utilisation in patients with CAD in the US, and may help decision makers optimise patient management by better understanding the impact of imaging modality choice on downstream diagnostic tests, interventions, health system encounters, and acute cardiac events.

## Conclusion

Choice of imaging modality for CAD diagnosis impacts downstream healthcare utilisation. Among patients in the US with suspected CAD who were diagnosed within 3 months of their index test, PET MPI was associated with lower downstream healthcare utilisation than other diagnostic modalities across multiple metrics, including follow-up testing and ICA. Efforts to improve clinician awareness of the strengths of PET MPI, in terms of its greater sensitivity for CAD detection and potential for reduced downstream healthcare utilisation, may help better inform referral decisions and guide post-imaging patient management.

## Supplementary Information

Supplementary Material 1.

## Data Availability

The data that support the findings of this study are available from Clarivate but restrictions apply to the availability of these data, which were used under license for the current study, and so are not publicly available. Data are however available from the authors upon reasonable request and with permission of Clarivate (Santosh.Angadageri@Clarivate.com).

## Abbreviations

ACC: American College of Cardiology
ACS: Acute coronary syndrome
AHA: American Heart Association
AHF: Acute heart failure
ASE: American Society of Echocardiograph
CABG: Coronary artery bypass graft
CAD: Coronary artery disease
cCTA: Coronary computed tomography angiography
CHEST: American College of Chest Physicians
CV: Coefficient of variation
ECG: Electrocardiogram
ER: Emergency room
ICA: Invasive coronary angiography
IS: Ischaemic stroke
MPI: Myocardial perfusion imaging
NICE: National Institute for Health and Care Excellence
NSTEMI: Non-ST-elevation myocardial infarction
PCI: Percutaneous coronary intervention
PET: Positron emission tomography myocardial
SAEM: Society for Academic Emergency Medicine
SCCT: Society of Cardiovascular Computed Tomography
SCMR: Society for Cardiovascular Magnetic Resonance
SD: Standard deviation
SPECT: Single-photon emission computed tomography
STEMI: ST-elevation myocardial infarction
TIA: Transient ischaemic event
UA: Unstable angina
