# Supplementary material for: Real-world evidence study on the impact of SPECT MPI, PET MPI, cCTA and stress echocardiography on downstream healthcare utilisation in patients with coronary artery disease in the US
Source: BMC Cardiovasc Disord. 2024 Oct 9;24:543. doi: 10.1186/s12872-024-04225-y (PMC11462745; doi:10.1186/s12872-024-04225-y)
Supplement: Supplementary file 1 — Supplementary Material 1. [file 12872_2024_4225_MOESM1_ESM.docx]

**Real-world evidence study on the impact of SPECT MPI, PET MPI, cCTA and stress echocardiography on downstream healthcare utilisation in patients with coronary artery disease in the US: Supplemental information**

[Figure S 1: Proxy pre-test CAD risk stratification method 2](#_Toc163205754)

[Figure S 2: Patient pathways by cohort and index test (Cohorts 1 & 2) 12](#_Toc163205755)

[Figure S 3: Patient pathways by cohort and index test (Cohorts 3 & 4) 13](#_Toc163205756)

[Figure S 4: Patient pathways by cohort and index test (Cohort 5) 14](#_Toc163205757)

[Figure S 5: Patient pathways by cohort and index test (Cohorts 6 & 7) 15](#_Toc163205758)

[Figure S 6: Patient pathways by cohort and index test (Cohorts 8 & 9) 16](#_Toc163205759)

[Figure S 7: Proportions of patients with any downstream healthcare utilisation in those who underwent combinations of imaging tests 17](#_Toc163205760)

[Table S 1: Downstream healthcare utilisation by patient cohort 2](#_Toc163205939)

Supporting information

Figure S 1: Proxy pre-test CAD risk stratification method


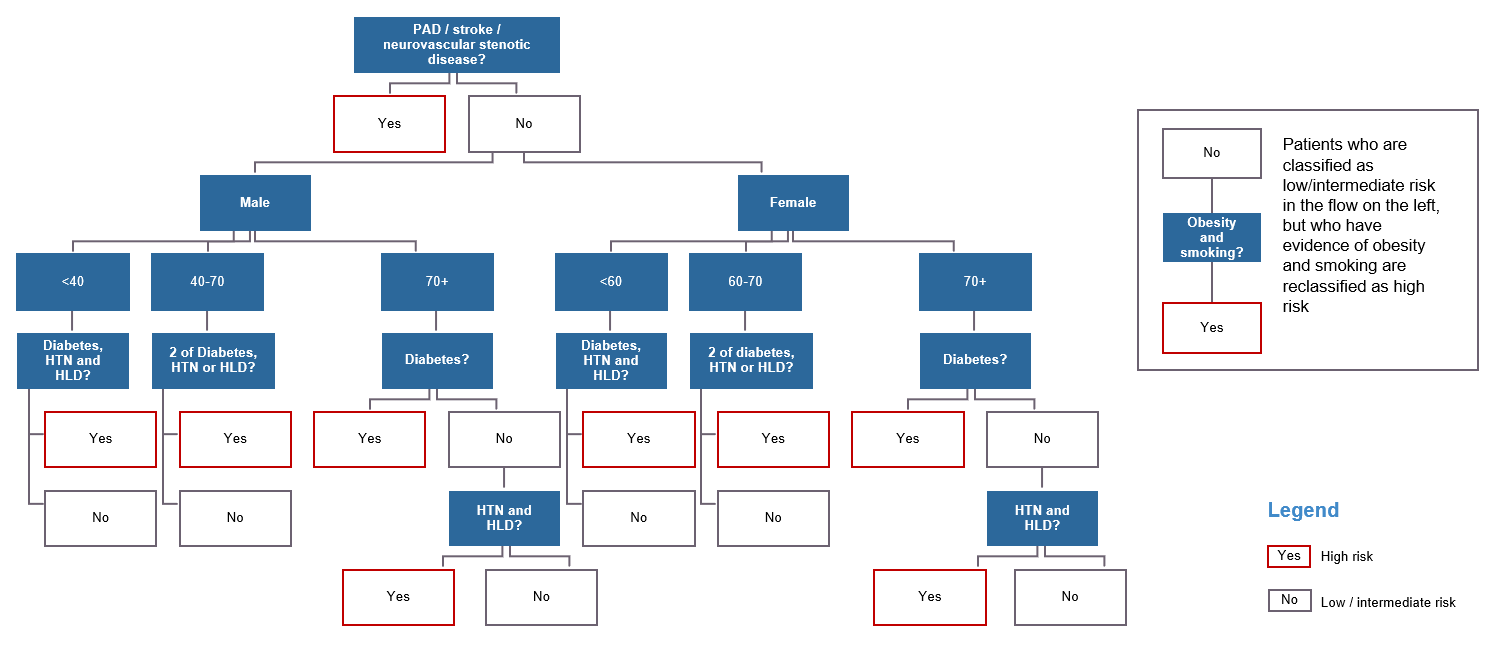


Abbreviations: HTN hypertension; HLD, hyperlipidaemia; PAD, peripheral arterial disease.

Table S 1: Downstream healthcare utilisation by patient cohort

| **Cohort 1 – Low pre-test risk, CAD diagnosis within 3 months of index test** | | | | | | | | | | | | |
| --- | --- | --- | --- | --- | --- | --- | --- | --- | --- | --- | --- | --- |
|  | **SPECT MPI** | | | **PET MPI** | | | **cCTA** | | | **SE** | | |
| **Additional diagnostic imaging** | | | | | | | | | | | | |
| Any imaging (%) | 3.5 | SE (%) | 9 | 1.7 | SE (%) | 6 | 8.0 | SE (%) | 13 | 4.2 | SE (%) | 36 |
|  |  | SPECT MPI (%) | 77 |  | SPECT MPI (%) | 38 |  | SPECT MPI (%) | 52 |  | SPECT MPI (%) | 40 |
|  |  | PET MPI (%) | 1 |  | PET MPI (%) | 25 |  | PET MPI (%) | 3 |  | PET MPI (%) | 5 |
|  |  | cCTA (%) | 12 |  | cCTA (%) | 31 |  | cCTA (%) | 32 |  | cCTA (%) | 19 |
| **Coronary angiography** (%) | 31.3 |  | | 15.4 |  | | 17.4 |  | | 31.0 |  | |
| **Interventions** | | | | | | | | | | | | |
| Total intervention (%) | 9.0 | PCI (%) | 86 | 5.8 | PCI (%) | 89 | 6.4 | PCI (%) | 92 | 10.1 | PCI (%) | 83 |
|  |  | CABG (%) | 14 |  | CABG (%) | 11 |  | CABG (%) | 8 |  | CABG (%) | 17 |
| **Cardiac-related health system encounters** | | | | | | | | | | | | |
| Hospital inpatient (%) | 3.4 |  | | 1.9 |  | | 5.3 |  | | 3.3 |  | |
| Hospital outpatient (%) | 4.4 |  |  | 2.6 |  |  | 4.2 |  |  | 5.4 |  |  |
| Cardiac office (%) | 5.8 |  |  | 6.2 |  |  | 4.2 |  |  | 4.7 |  |  |
| Emergency (%) | 1.5 |  |  | 0.8 |  |  | 1.2 |  |  | 1.1 |  |  |
| **Events** | | | | | | | | | | | | |
| Any event (%) | 9.5 | IS (%) | 9 | 5.3 | IS (%) | 12 | 6.9 | IS (%) | 10 | 8.6 | IS (%) | 6 |
|  |  | TIA (%) | 1 |  | TIA (%) | 1 |  | TIA (%) | 0 |  | TIA (%) | 1 |
|  |  | AHF (%) | 13 |  | AHF (%) | 20 |  | AHF (%) | 13 |  | AHF (%) | 6 |
|  |  | ACS (%) | 77 |  | ACS (%) | 67 |  | ACS (%) | 77 |  | ACS (%) | 86 |

| **Cohort 2 – Low pre-test risk, no CAD diagnosis within 3 months of index test** | | | | | | | | | | | | |
| --- | --- | --- | --- | --- | --- | --- | --- | --- | --- | --- | --- | --- |
|  | **SPECT MPI** | | | **PET MPI** | | | **cCTA** | | | **SE** | | |
| **Additional diagnostic imaging** | | | | | | | | | | | | |
| Any imaging (%) | 2.1 | SE (%) | 9 | 1.3 | SE (%) | 10 | 3.6 | SE (%) | 19 | 1.8% | SE (%) | 56 |
|  |  | SPECT MPI (%) | 81 |  | SPECT MPI (%) | 33 |  | SPECT MPI (%) | 36 |  | SPECT MPI (%) | 30 |
|  |  | PET MPI (%) | 1 |  | PET MPI (%) | 37 |  | PET MPI (%) | 2 |  | PET MPI (%) | 2 |
|  |  | cCTA (%) | 9 |  | cCTA (%) | 20 |  | cCTA (%) | 44 |  | cCTA (%) | 12 |
| **Coronary angiography** (%) | 2.9 |  | | 2.2 |  | | 1.1 |  | | 1.3 |  | |
| **Interventions** | | | | | | | | | | | | |
| Total intervention (%) | Nil | PCI (%) | Nil | Nil | PCI (%) | Nil | Nil | PCI (%) | Nil | Nil | PCI (%) | Nil |
|  |  | CABG (%) | Nil |  | CABG (%) | Nil |  | CABG (%) | Nil |  | CABG (%) | Nil |
| **Cardiac-related health system encounters** | | | | | | | | | | | | |
| Hospital inpatient (%) | 1.1 |  | | 1.0 |  | | 2.4 |  | | 0.6 |  | |
| Hospital outpatient (%) | 2.1 |  |  | 2.5 |  |  | 3.0 |  |  | 1.8 |  |  |
| Cardiac office (%) | 3.9 |  |  | 6.1 |  |  | 3.3 |  |  | 3.0 |  |  |
| Emergency (%) | 0.7 |  |  | 0.5 |  |  | 0.9 |  |  | 0.4 |  |  |
| **Events** | | | | | | | | | | | | |
| Any event (%) | 1.6 | IS (%) | 35 | 1.7 | IS (%) | 29 | 1.2 | IS (%) | 32 | 0.6 | IS (%) | 44 |
|  |  | TIA (%) | 4 |  | TIA (%) | 2 |  | TIA (%) | 2 |  | TIA (%) | 7 |
|  |  | AHF (%) | 26 |  | AHF (%) | 42 |  | AHF (%) | 30 |  | AHF (%) | 13 |
|  |  | ACS (%) | 34 |  | ACS (%) | 27 |  | ACS (%) | 36 |  | ACS (%) | 36 |

| **Cohort 3 – High pre-test risk, CAD diagnosis within 3 months of index test** | | | | | | | | | | | | |
| --- | --- | --- | --- | --- | --- | --- | --- | --- | --- | --- | --- | --- |
|  | **SPECT MPI** | | | **PET MPI** | | | **cCTA** | | | **SE** | | |
| **Additional diagnostic imaging** | | | | | | | | | | | | |
| Any imaging (%) | 4.7 | SE (%) | 8 | 2.8 | SE (%) | 8 | 10.1 | SE (%) | 11 | 5.0 | SE (%) | 41 |
|  |  | SPECT MPI (%) | 82 |  | SPECT MPI (%) | 36 |  | SPECT MPI (%) | 57 |  | SPECT MPI (%) | 42 |
|  |  | PET MPI (%) | 1 |  | PET MPI (%) | 31 |  | PET MPI (%) | 2 |  | PET MPI (%) | 4 |
|  |  | cCTA (%) | 9 |  | cCTA (%) | 25 |  | cCTA (%) | 29 |  | cCTA (%) | 13 |
| **Coronary angiography** (%) | 38.2 |  | | 24.2 |  | | 21.5 |  | | 36.5 |  | |
| **Interventions** | | | | | | | | | | | | |
| Total intervention (%) | 11.9 | PCI (%) | 83 | 9.6 | PCI (%) | 85 | 8.4 | PCI (%) | 85 | 13.1 | PCI (%) | 79 |
|  |  | CABG (%) | 17 |  | CABG (%) | 15 |  | CABG (%) | 15 |  | CABG (%) | 21 |
| **Cardiac-related health system encounters** | | | | | | | | | | | | |
| Hospital inpatient (%) | 5.7 |  | | 4.0 |  | | 7.5 |  | | 5.3 |  | |
| Hospital outpatient (%) | 5.3 |  |  | 4.1 |  |  | 5.2 |  |  | 6.6 |  |  |
| Cardiac office (%) | 5.6 |  |  | 7.2 |  |  | 4.7 |  |  | 4.7 |  |  |
| Emergency (%) | 2.5 |  |  | 2.0 |  |  | 2.1 |  |  | 1.9 |  |  |
| **Events** | | | | | | | | | | | | |
| Any event (%) | 13.2 | IS (%) | 14 | 9.4 | IS (%) | 18 | 9.9 | IS (%) | 16 | 12.3 | IS (%) | 9 |
|  |  | TIA (%) | 1 |  | TIA (%) | 2 |  | TIA (%) | 1 |  | TIA (%) | 1 |
|  |  | AHF (%) | 13 |  | AHF (%) | 22 |  | AHF (%) | 13 |  | AHF (%) | 7 |
|  |  | ACS (%) | 71 |  | ACS (%) | 58 |  | ACS (%) | 71 |  | ACS (%) | 83 |

| **Cohort 4 – High pre-test risk, no CAD diagnosis within 3 months of index test** | | | | | | | | | | | | |
| --- | --- | --- | --- | --- | --- | --- | --- | --- | --- | --- | --- | --- |
|  | **SPECT MPI** | | | **PET MPI** | | | **cCTA** | | | **SE** | | |
| **Additional diagnostic imaging** | | | | | | | | | | | | |
| Any imaging (%) | 2.6 | SE (%) | 8 | 1.7 | SE (%) | 10 | 4.4 | SE (%) | 15 | 2.4 | SE (%) | 54 |
|  |  | SPECT MPI (%) | 86 |  | SPECT MPI (%) | 34 |  | SPECT MPI (%) | 45 |  | SPECT MPI (%) | 36 |
|  |  | PET MPI (%) | 1 |  | PET MPI (%) | 39 |  | PET MPI (%) | 1 |  | PET MPI (%) | 3 |
|  |  | cCTA (%) | 5 |  | cCTA (%) | 17 |  | cCTA (%) | 39 |  | cCTA (%) | 7 |
| **Coronary angiography** (%) | 3.5 |  | | 2.9 |  | | 1.6 |  | | 1.6 |  | |
| **Interventions** | | | | | | | | | | | | |
| Total intervention (%) | Nil | PCI (%) | Nil | Nil | PCI (%) | Nil | Nil | PCI (%) | Nil | Nil | PCI (%) | Nil |
|  |  | CABG (%) | Nil |  | CABG (%) | Nil |  | CABG (%) | Nil |  | CABG (%) | Nil |
| **Cardiac-related health system encounters** | | | | | | | | | | | | |
| Hospital inpatient (%) | 1.8 |  | | 1.8 |  | | 4.2 |  | | 1.1 |  | |
| Hospital outpatient (%) | 2.2 |  |  | 2.6 |  |  | 3.2 |  |  | 1.9 |  |  |
| Cardiac office (%) | 3.5 |  |  | 4.9 |  |  | 3.9 |  |  | 2.7 |  |  |
| Emergency (%) | 1.1 |  |  | 0.9 |  |  | 1.2 |  |  | 0.7 |  |  |
| **Events** | | | | | | | | | | | | |
| Any event (%) | 2.8 | IS (%) | 46 | 3.1 | IS (%) | 41 | 2.9 | IS (%) | 45 | 1.4 | IS (%) | 54 |
|  |  | TIA (%) | 5 |  | TIA (%) | 2 |  | TIA (%) | 4 |  | TIA (%) | 7 |
|  |  | AHF (%) | 24 |  | AHF (%) | 36 |  | AHF (%) | 29 |  | AHF (%) | 14 |
|  |  | ACS (%) | 26 |  | ACS (%) | 21 |  | ACS (%) | 22 |  | ACS (%) | 25 |

| **Cohort 5 – Existing CAD diagnosis, no prior cardiac events** | | | | | | | | | | | | |
| --- | --- | --- | --- | --- | --- | --- | --- | --- | --- | --- | --- | --- |
|  | **SPECT MPI** | | | **PET MPI** | | | **cCTA** | | | **SE** | | |
| **Additional diagnostic imaging** | | | | | | | | | | | | |
| Any imaging (%) | 3.5 | SE (%) | 7 | 1.9 | SE (%) | 6 | 7.3 | SE (%) | 12 | 3.8 | SE (%) | 43 |
|  |  | SPECT MPI (%) | 87 |  | SPECT MPI (%) | 51 |  | SPECT MPI (%) | 55 |  | SPECT MPI (%) | 48 |
|  |  | PET MPI (%) | 1 |  | PET MPI (%) | 30 |  | PET MPI (%) | 3 |  | PET MPI (%) | 3 |
|  |  | cCTA (%) | 5 |  | cCTA (%) | 12 |  | cCTA (%) | 30 |  | cCTA (%) | 7 |
| **Coronary angiography** (%) | 15 |  | | 14.9 |  | | 15.9 |  | | 9.9 |  | |
| **Interventions** | | | | | | | | | | | | |
| Total intervention (%) | 5.3 | PCI (%) | 91 | 6.8 | PCI (%) | 93 | 6.2 | PCI (%) | 88 | 3.6 | PCI (%) | 86 |
|  |  | CABG (%) | 9 |  | CABG (%) | 7 |  | CABG (%) | 12 |  | CABG (%) | 14 |
| **Cardiac-related health system encounters** | | | | | | | | | | | | |
| Hospital inpatient (%) | 4.1 |  | | 3.5 |  | | 13.6 |  | | 3.0 |  | |
| Hospital outpatient (%) | 4.4 |  |  | 3.8 |  |  | 8.9 |  |  | 3.7 |  |  |
| Cardiac office (%) | 7.6 |  |  | 11.0 |  |  | 9.3 |  |  | 5.1 |  |  |
| Emergency (%) | 2.3 |  |  | 1.9 |  |  | 2.9 |  |  | 1.5 |  |  |
| **Events** | | | | | | | | | | | | |
| Any event (%) | 8.0 | IS (%) | 17 | 7.7 | IS (%) | 16 | 9.0 | IS (%) | 19 | 5.1 | IS (%) | 17 |
|  |  | TIA (%) | 2 |  | TIA (%) | 2 |  | TIA (%) | 1 |  | TIA (%) | 2 |
|  |  | AHF (%) | 20 |  | AHF (%) | 24 |  | AHF (%) | 24 |  | AHF (%) | 15 |
|  |  | ACS (%) | 62 |  | ACS (%) | 58 |  | ACS (%) | 56 |  | ACS (%) | 66 |

| **Cohort 6 – Existing CAD diagnosis, recent prior event (0–1 year pre-index)** | | | | | | | | | | | | |
| --- | --- | --- | --- | --- | --- | --- | --- | --- | --- | --- | --- | --- |
|  | **SPECT MPI** | | | **PET MPI** | | | **cCTA** | | | **SE** | | |
| **Additional diagnostic imaging** | | | | | | | | | | | | |
| Any imaging (%) | 7.6 | SE (%) | 7 | 6.2 | SE (%) | 8 | 10.3 | SE (%) | 12 | 8.8 | SE (%) | 37 |
|  |  | SPECT MPI (%) | 89 |  | SPECT MPI (%) | 46 |  | SPECT MPI (%) | 58 |  | SPECT MPI (%) | 56 |
|  |  | PET MPI (%) | 2 |  | PET MPI (%) | 40 |  | PET MPI (%) | 5 |  | PET MPI (%) | 2 |
|  |  | cCTA (%) | 2 |  | cCTA (%) | 6 |  | cCTA (%) | 26 |  | cCTA (%) | 5 |
| **Coronary angiography** (%) | 23.8 |  | | 27.1 |  | | 22.1 |  | | 18.2 |  | |
| **Interventions** | | | | | | | | | | | | |
| Total intervention (%) | 11.4 | PCI (%) | 90 | 16.3 | PCI (%) | 86 | 12.5 | PCI (%) | 86 | 8.5 | PCI (%) | 86 |
|  |  | CABG (%) | 10 |  | CABG (%) | 14 |  | CABG (%) | 14 |  | CABG (%) | 14 |
| **Cardiac-related health system encounters** | | | | | | | | | | | | |
| Hospital inpatient (%) | 12.2 |  | | 13.6 |  | | 27.5 |  | | 10.4 |  | |
| Hospital outpatient (%) | 8.5 |  |  | 11.4 |  |  | 17.4 |  |  | 9.7 |  |  |
| Cardiac office (%) | 8.7 |  |  | 12.2 |  |  | 13.7 |  |  | 7.5 |  |  |
| Emergency (%) | 8.0 |  |  | 7.6 |  |  | 8.3 |  |  | 5.5 |  |  |
| **Events** | | | | | | | | | | | | |
| Any event (%) | 31.9 | IS (%) | 15 | 36.8 | IS (%) | 11 | 36.0 | IS (%) | 16 | 25.0 | IS (%) | 14 |
|  |  | TIA (%) | 1 |  | TIA (%) | 1 |  | TIA (%) | 1 |  | TIA (%) | 1 |
|  |  | AHF (%) | 31 |  | AHF (%) | 41 |  | AHF (%) | 32 |  | AHF (%) | 28 |
|  |  | ACS (%) | 53 |  | ACS (%) | 47 |  | ACS (%) | 52 |  | ACS (%) | 57 |

| **Cohort 7 – Existing CAD diagnosis, recent prior event (1–2 years pre-index)** | | | | | | | | | | | | |
| --- | --- | --- | --- | --- | --- | --- | --- | --- | --- | --- | --- | --- |
|  | **SPECT MPI** | | | **PET MPI** | | | **cCTA** | | | **SE** | | |
| **Additional diagnostic imaging** | | | | | | | | | | | | |
| Any imaging (%) | 5.7 | SE (%) | 6 | 3.1 | SE (%) | 5 | 12.4 | SE (%) | 14 | 5.8 | SE (%) | 45 |
|  |  | SPECT MPI (%) | 90 |  | SPECT MPI (%) | 48 |  | SPECT MPI (%) | 53 |  | SPECT MPI (%) | 49 |
|  |  | PET MPI (%) | 1 |  | PET MPI (%) | 44 |  | PET MPI (%) | 3 |  | PET MPI (%) | 3 |
|  |  | cCTA (%) | 2 |  | cCTA (%) | 3 |  | cCTA (%) | 30 |  | cCTA (%) | 3 |
| **Coronary angiography** (%) | 19.7 |  | | 19.6 |  | | 19.8 |  | | 14.3 |  | |
| **Interventions** | | | | | | | | | | | | |
| Total intervention (%) | 8.0 | PCI (%) | 93 | 8.8 | PCI (%) | 89 | 8.5 | PCI (%) | 94 | 5.4 | PCI (%) | 88 |
|  |  | CABG (%) | 7 |  | CABG (%) | 11 |  | CABG (%) | 6 |  | CABG (%) | 12 |
| **Cardiac-related health system encounters** | | | | | | | | | | | | |
| Hospital inpatient (%) | 7.6 |  | | 7.8 |  | | 25.5 |  | | 5.7 |  | |
| Hospital outpatient (%) | 6.3 |  |  | 7.7 |  |  | 14.1 |  |  | 5.4 |  |  |
| Cardiac office (%) | 8.4 |  |  | 15.2 |  |  | 11.6 |  |  | 6.0 |  |  |
| Emergency (%) | 4.9 |  |  | 4.5 |  |  | 6.8 |  |  | 3.5 |  |  |
| **Events** | | | | | | | | | | | | |
| Any event (%) | 16.8 | IS (%) | 19 | 17.9 | IS (%) | 15 | 21.4 | IS (%) | 27 | 10.9 | IS (%) | 19 |
|  |  | TIA (%) | 2 |  | TIA (%) | 1 |  | TIA (%) | 2 |  | TIA (%) | 2 |
|  |  | AHF (%) | 24 |  | AHF (%) | 29 |  | AHF (%) | 31 |  | AHF (%) | 20 |
|  |  | ACS (%) | 55 |  | ACS (%) | 54 |  | ACS (%) | 39 |  | ACS (%) | 60 |

| **Cohort 8 – Low pre-test risk, subsequent CAD diagnosis >3 months after index test** | | | | | | | | | | | | |
| --- | --- | --- | --- | --- | --- | --- | --- | --- | --- | --- | --- | --- |
|  | **SPECT MPI** | | | **PET MPI** | | | **cCTA** | | | **SE** | | |
| **Additional diagnostic imaging** | | | | | | | | | | | | |
| Any imaging (%) | 4.8 | SE (%) | 9 | 2.7 | SE (%) | 2 | 6.7 | SE (%) | 15 | 6.1 | SE (%) | 29 |
|  |  | SPECT MPI (%) | 71 |  | SPECT MPI (%) | 47 |  | SPECT MPI (%) | 49 |  | SPECT MPI (%) | 41 |
|  |  | PET MPI (%) | 2 |  | PET MPI (%) | 26 |  | PET MPI (%) | 4 |  | PET MPI (%) | 3 |
|  |  | cCTA (%) | 19 |  | cCTA (%) | 26 |  | cCTA (%) | 32 |  | cCTA (%) | 28 |
| **Coronary angiography** (%) | 13.1 |  | | 9.0 |  | | 6.6 |  | | 10.8 |  | |
| **Interventions** | | | | | | | | | | | | |
| Total intervention (%) | 2.7 | PCI (%) | 91 | 2.8 | PCI (%) | 90 | 1.0 | PCI (%) | 91 | 2.5 | PCI (%) | 88 |
|  |  | CABG (%) | 9 |  | CABG (%) | 10 |  | CABG (%) | 9 |  | CABG (%) | 12 |
| **Cardiac-related health system encounters** | | | | | | | | | | | | |
| Hospital inpatient (%) | 3.7 |  | | 4.1 |  | | 5.4 |  | | 2.8 |  | |
| Hospital outpatient (%) | 4.4 |  |  | 4.8 |  |  | 5.9 |  |  | 4.0 |  |  |
| Cardiac office (%) | 6.6 |  |  | 8.5 |  |  | 6.2 |  |  | 5.0 |  |  |
| Emergency (%) | 2.1 |  |  | 2.3 |  |  | 2.0 |  |  | 1.6 |  |  |
| **Events** | | | | | | | | | | | | |
| Any event (%) | 7.5 | IS (%) | 16 | 6.8 | IS (%) | 16 | 5.0 | IS (%) | 10 | 5.1 | IS (%) | 13 |
|  |  | TIA (%) | 2 |  | TIA (%) | 2 |  | TIA (%) | 0 |  | TIA (%) | 3 |
|  |  | AHF (%) | 22 |  | AHF (%) | 36 |  | AHF (%) | 34 |  | AHF (%) | 10 |
|  |  | ACS (%) | 61 |  | ACS (%) | 46 |  | ACS (%) | 55 |  | ACS (%) | 73 |

| **Cohort 9 – High pre-test risk, subsequent CAD diagnosis >3 months after index test** | | | | | | | | | | | | |
| --- | --- | --- | --- | --- | --- | --- | --- | --- | --- | --- | --- | --- |
|  | **SPECT MPI** | | | **PET MPI** | | | **cCTA** | | | **SE** | | |
| **Additional diagnostic imaging** | | | | | | | | | | | | |
| Any imaging (%) | 6.0 | SE (%) | 8 | 4.0 | SE (%) | 11 | 9.1 | SE (%) | 10 | 7.1 | SE (%) | 30 |
|  |  | SPECT MPI (%) | 78 |  | SPECT MPI (%) | 44 |  | SPECT MPI (%) | 58 |  | SPECT MPI (%) | 49 |
|  |  | PET MPI (%) | 2 |  | PET MPI (%) | 26 |  | PET MPI (%) | 3 |  | PET MPI (%) | 3 |
|  |  | cCTA (%) | 13 |  | cCTA (%) | 19 |  | cCTA (%) | 29 |  | cCTA (%) | 18 |
| **Coronary angiography** (%) | 14.1 |  | | 11.5 |  | | 8.6 |  | | 11.9 |  | |
| **Interventions** | | | | | | | | | | | | |
| Total intervention (%) | 3.2 | PCI (%) | 87 | 2.6 | PCI (%) | 87 | 1.3 | PCI (%) | 89 | 3.2 | PCI (%) | 87 |
|  |  | CABG (%) | 13 |  | CABG (%) | 13 |  | CABG (%) | 11 |  | CABG (%) | 13 |
| **Cardiac-related health system encounters** | | | | | | | | | | | | |
| Hospital inpatient (%) | 5.7 |  | | 4.9 |  | | 8.2 |  | | 4.5 |  | |
| Hospital outpatient (%) | 4.6 |  |  | 4.7 |  |  | 7.1 |  |  | 4.3 |  |  |
| Cardiac office (%) | 5.9 |  |  | 7.6 |  |  | 7.5 |  |  | 4.5 |  |  |
| Emergency (%) | 3.6 |  |  | 2.1 |  |  | 3.4 |  |  | 2.7 |  |  |
| **Events** | | | | | | | | | | | | |
| Any event (%) | 10.0 | IS (%) | 24 | 8.4 | IS (%) | 26 | 7.3 | IS (%) | 27 | 7.4 | IS (%) | 22 |
|  |  | TIA (%) | 3 |  | TIA (%) | 1 |  | TIA (%) | 2 |  | TIA (%) | 3 |
|  |  | AHF (%) | 22 |  | AHF (%) | 34 |  | AHF (%) | 25 |  | AHF (%) | 15 |
|  |  | ACS (%) | 52 |  | ACS (%) | 39 |  | ACS (%) | 46 |  | ACS (%) | 60 |

Abbreviations: ACS, acute coronary syndrome; AHF, acute heart failure; CABG, coronary artery bypass graft; cCTA, coronary computed tomography angiography; IS, ischaemic stroke; PET MPI, positron emission tomography myocardial perfusion imaging; PCI, percutaneous coronary intervention; SE, stress echocardiography; SPECT MPI, single-photon emission computed tomography myocardial perfusion imaging; TIA, transient ischaemic event.

Figure S 2: Patient pathways by cohort and index test (Cohorts 1 & 2)


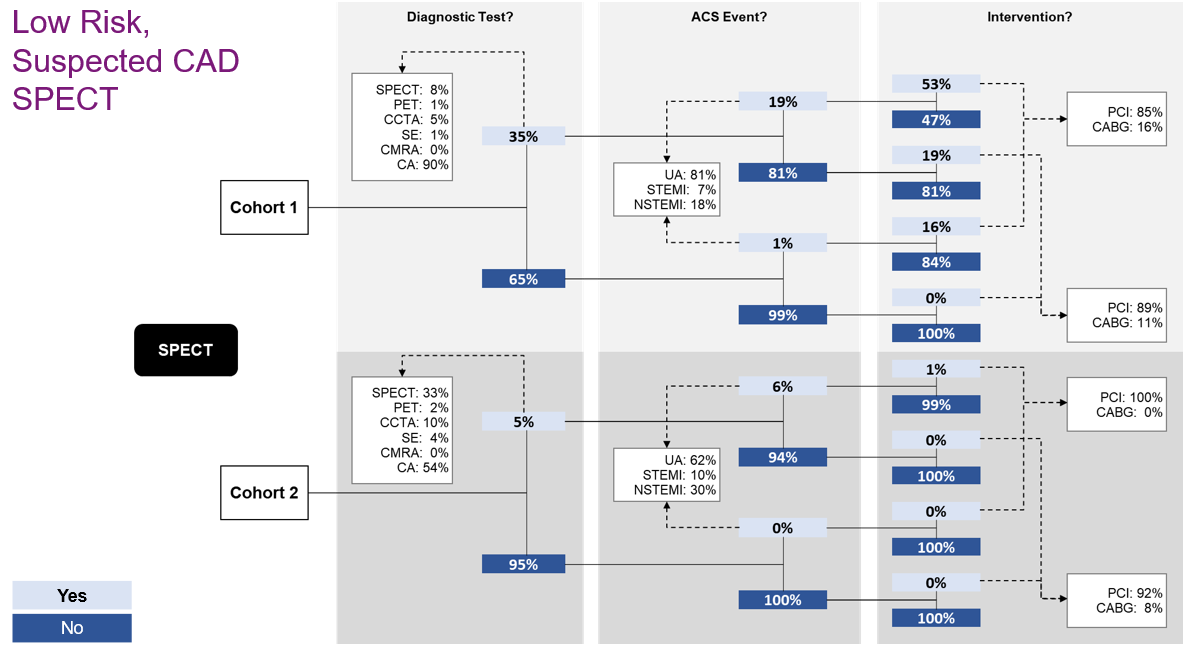

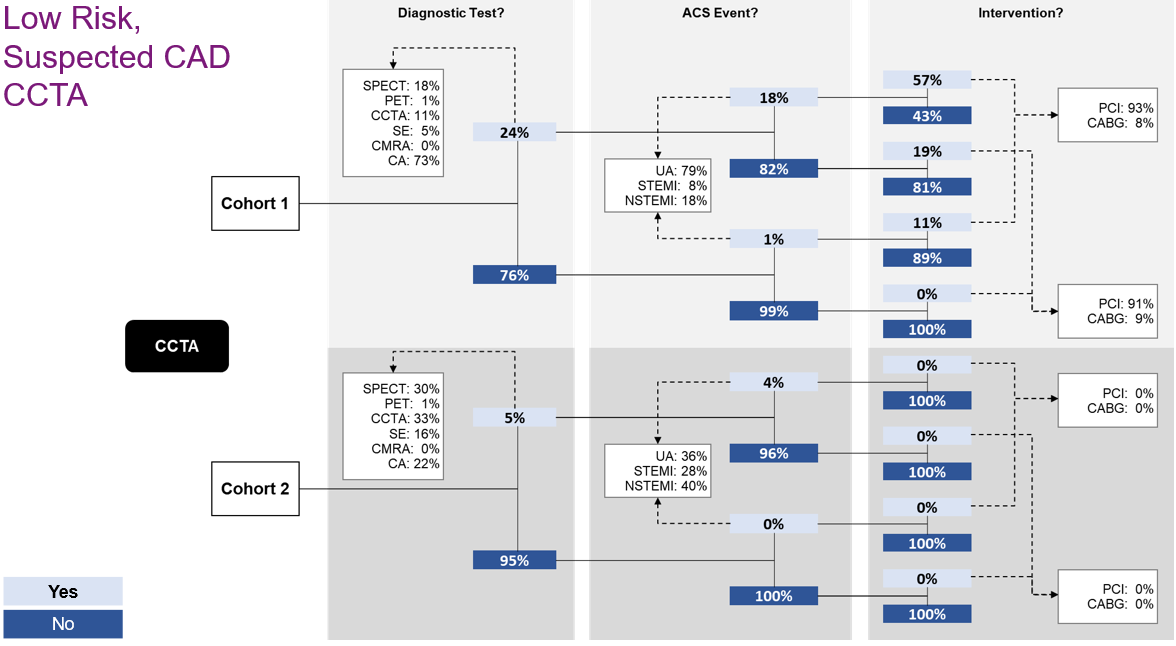


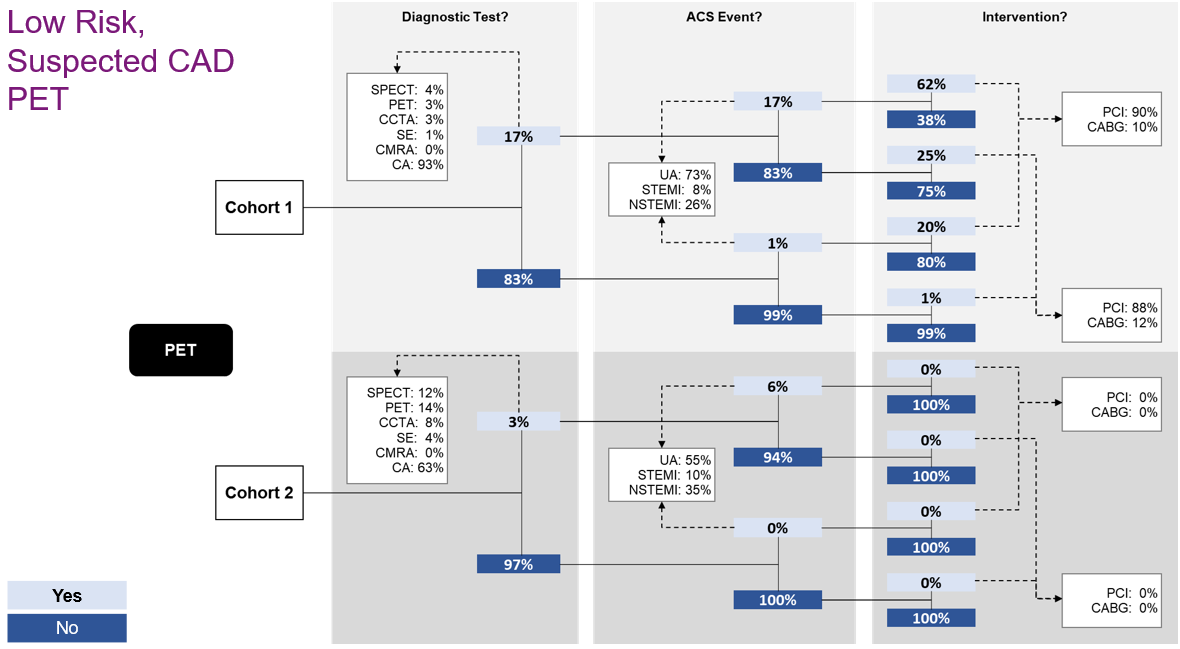

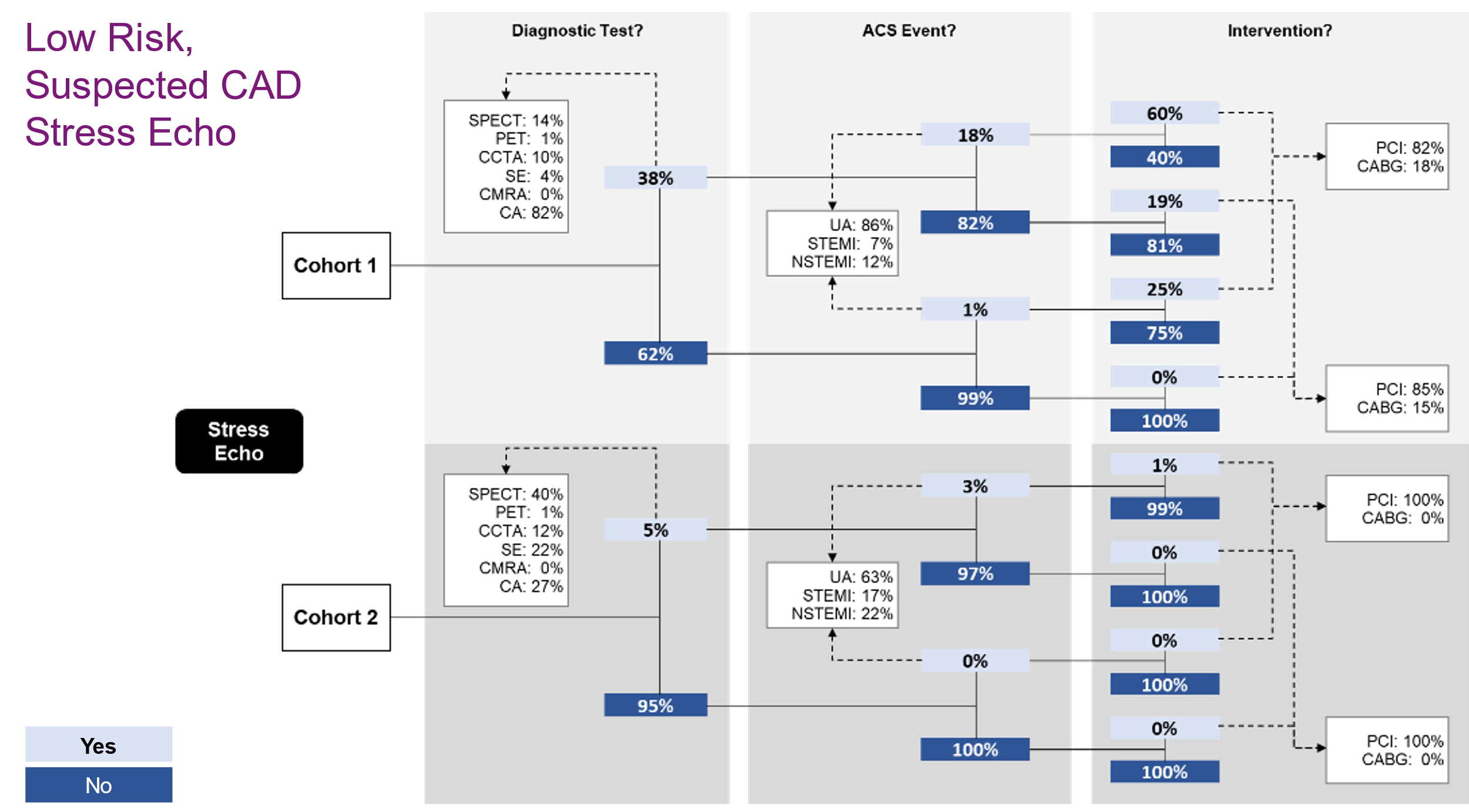


Abbreviations: ACS, acute coronary syndrome; CA, invasive coronary angiography; CABG, coronary artery bypass graft; CCTA, coronary computed tomography angiography; CMRA, coronary MR angiography; NSTEMI, non-ST-elevation myocardial infarction; PCI, percutaneous coronary intervention; PET, positron emission tomography; SE, stress echocardiography; SPECT, single-photon emission computed tomography; STEMI, ST-elevation myocardial infarction; UA, unstable angina.

Figure S 3: Patient pathways by cohort and index test (Cohorts 3 & 4)


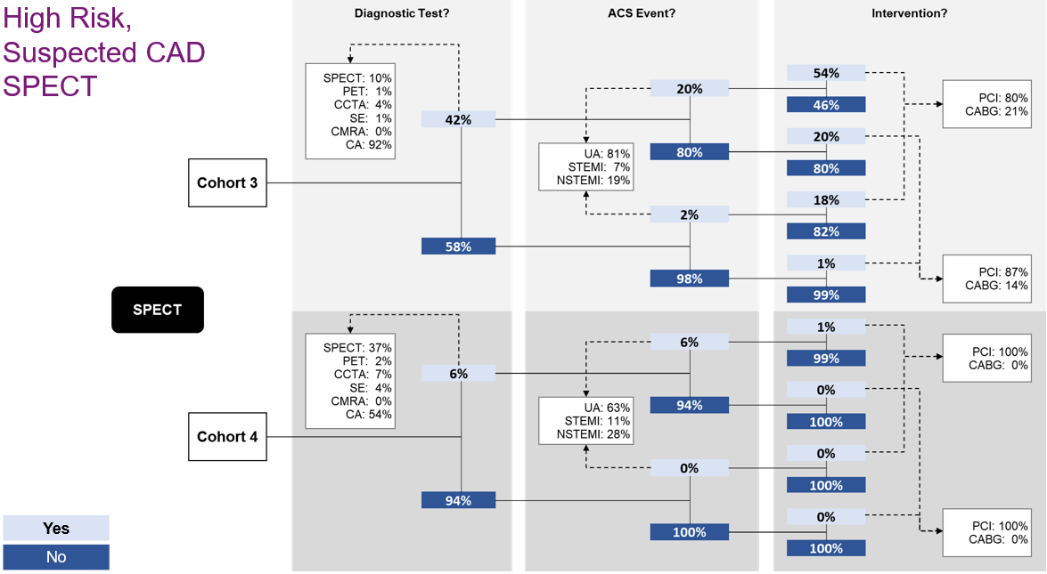

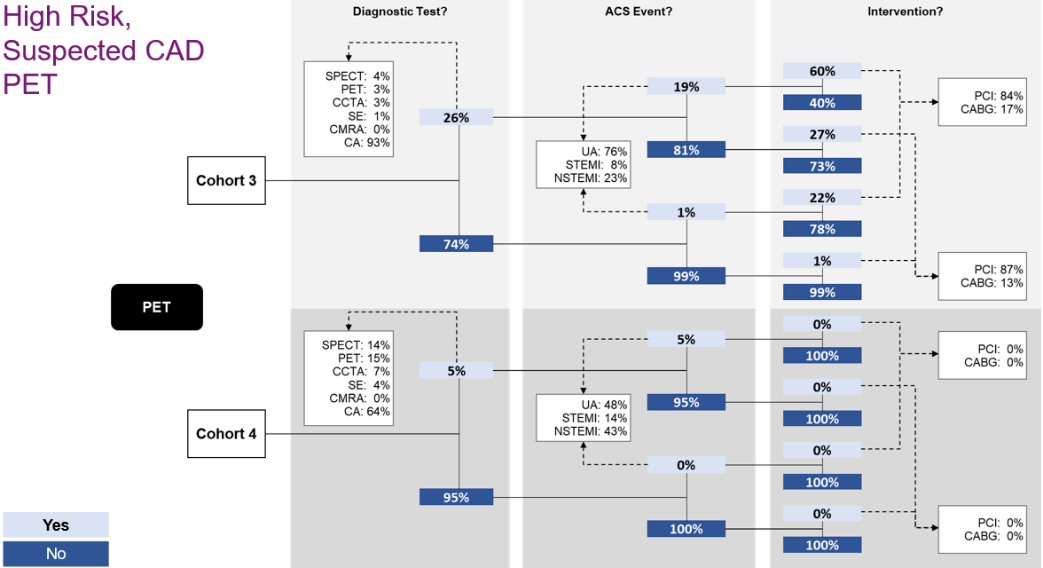


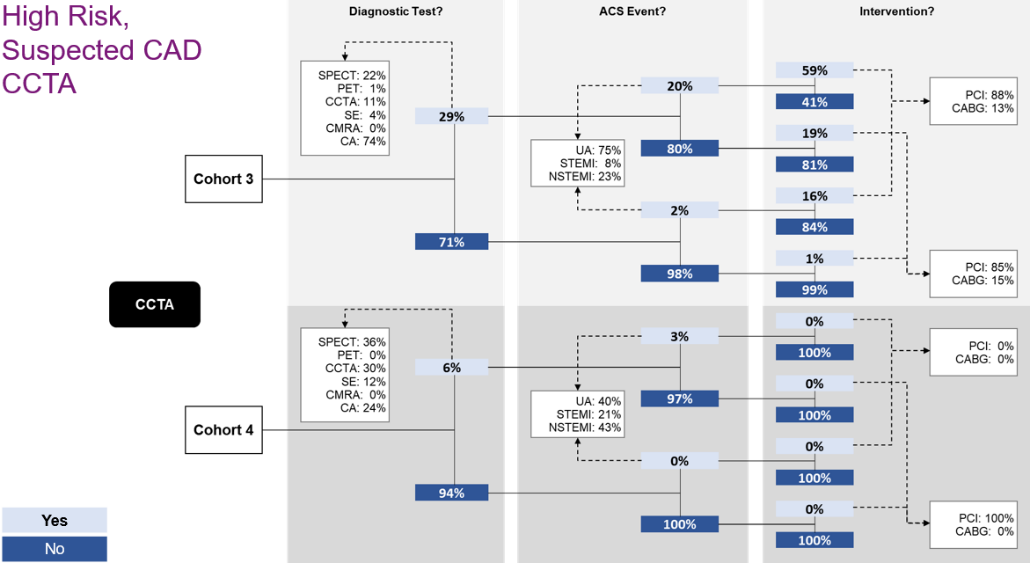

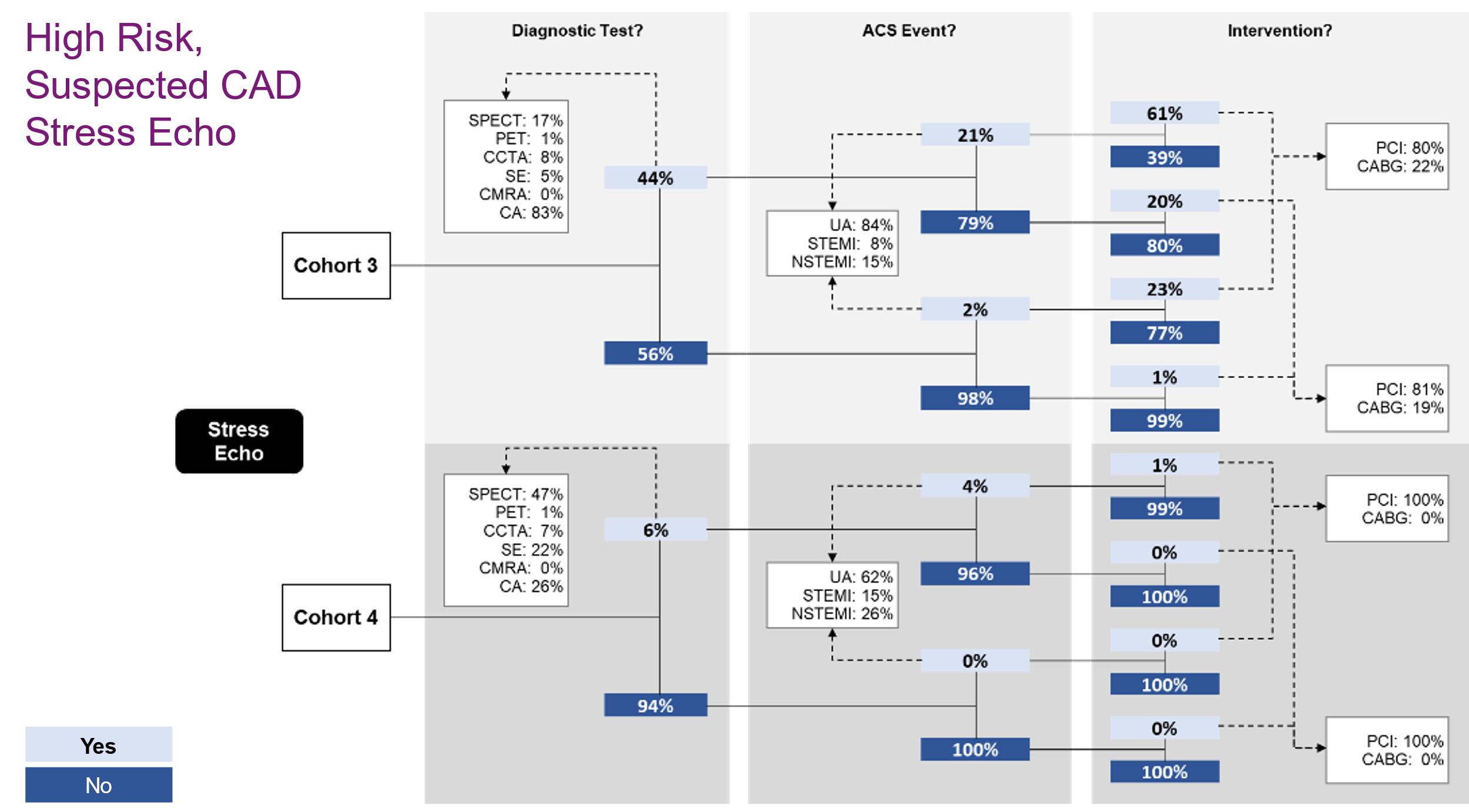


Abbreviations: ACS, acute coronary syndrome; CA, invasive coronary angiography; CABG, coronary artery bypass graft; CCTA, coronary computed tomography angiography; CMRA, coronary MR angiography; NSTEMI, non-ST-elevation myocardial infarction; PCI, percutaneous coronary intervention; PET, positron emission tomography; SE, stress echocardiography; SPECT, single-photon emission computed tomography; STEMI, ST-elevation myocardial infarction; UA, unstable angina.

Figure S 4: Patient pathways by cohort and index test (Cohort 5)


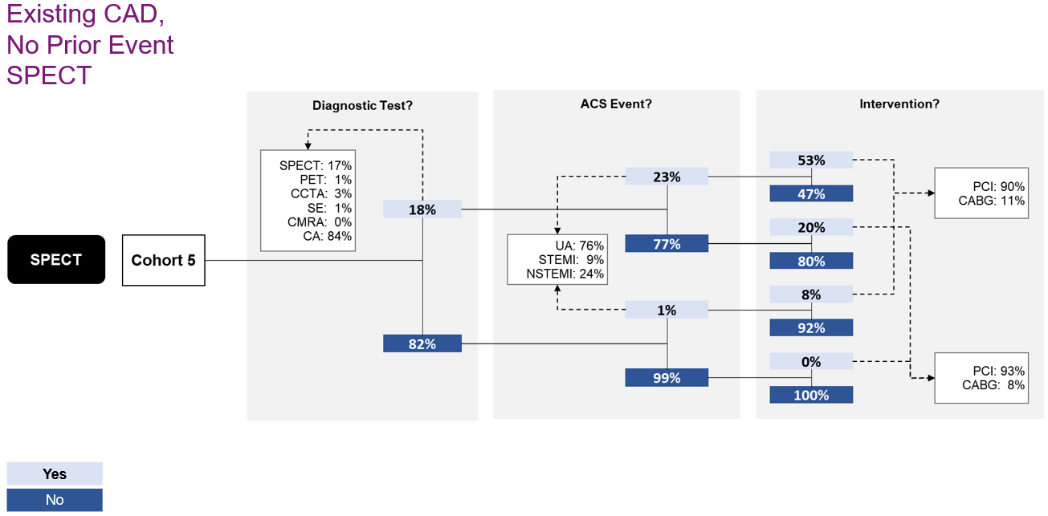

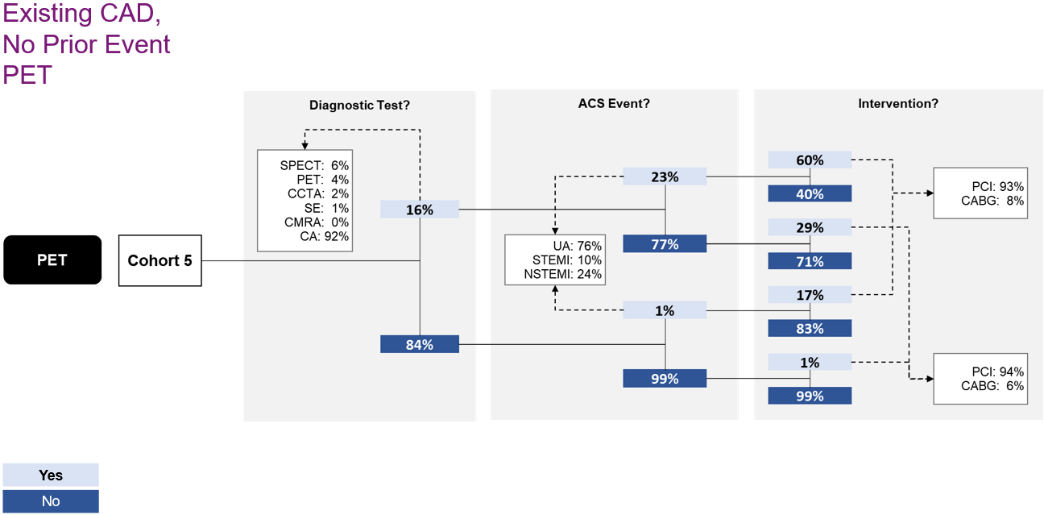


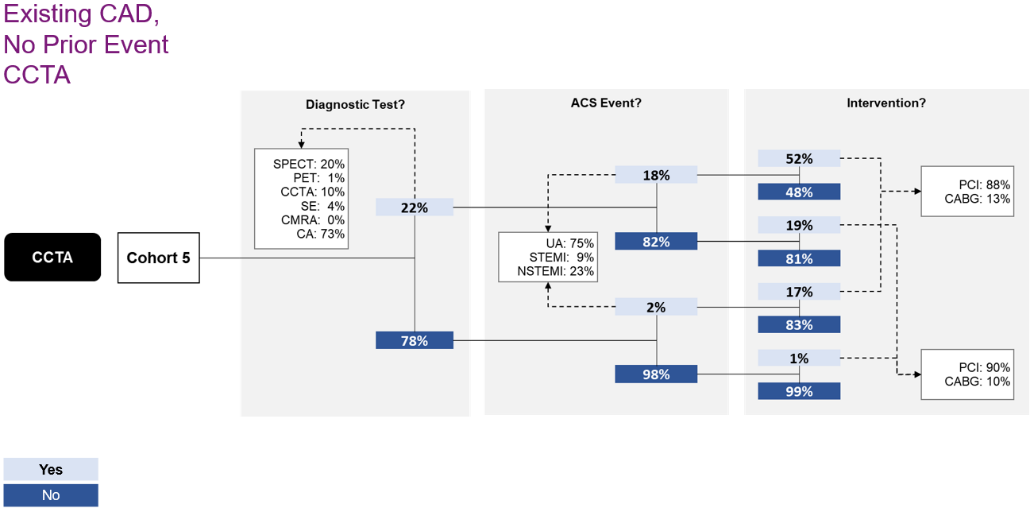

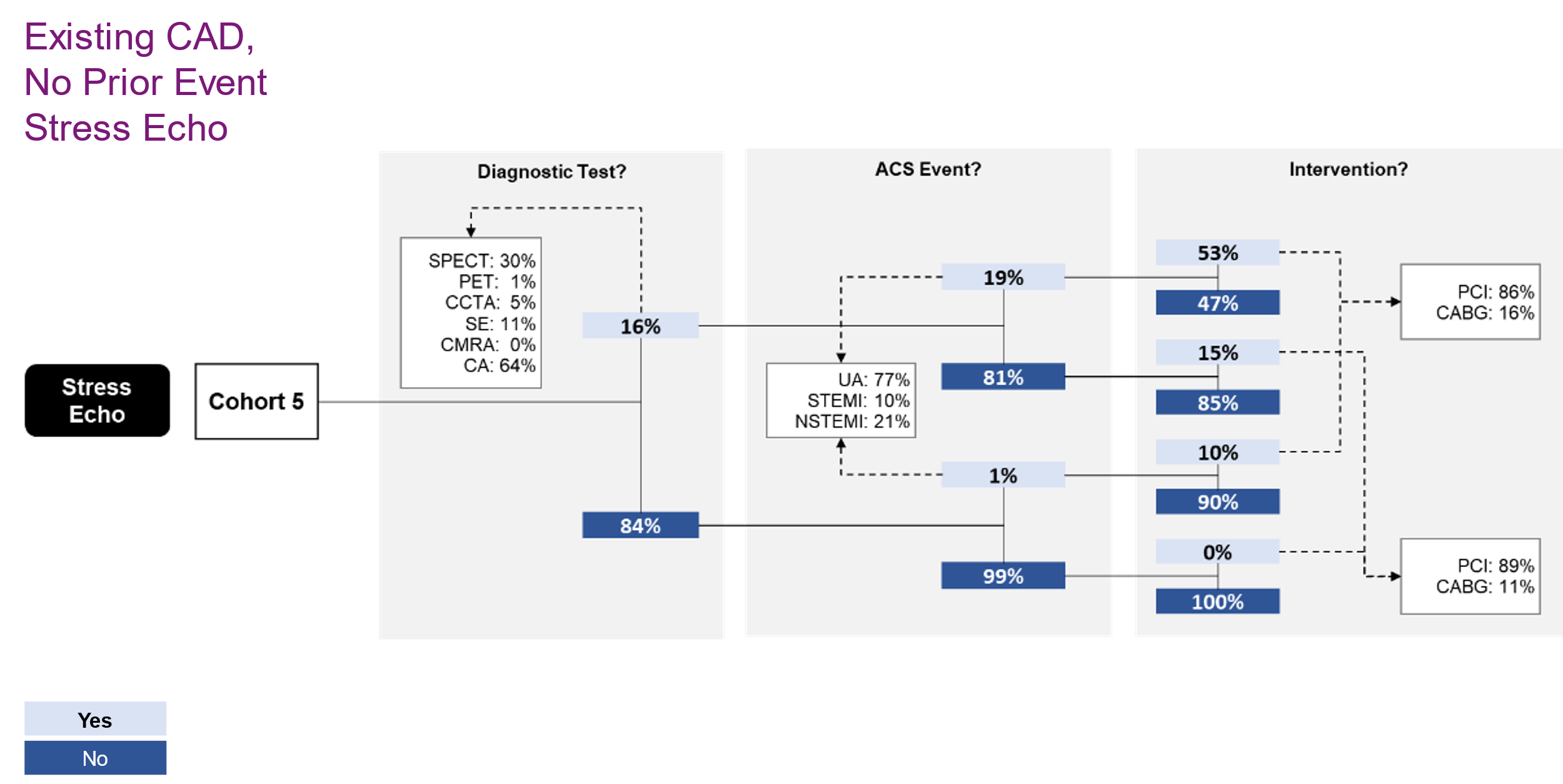


Abbreviations: ACS, acute coronary syndrome; CA, invasive coronary angiography; CABG, coronary artery bypass graft; CCTA, coronary computed tomography angiography; CMRA, coronary MR angiography; NSTEMI, non-ST-elevation myocardial infarction; PCI, percutaneous coronary intervention; PET, positron emission tomography; SE, stress echocardiography; SPECT, single-photon emission computed tomography; STEMI, ST-elevation myocardial infarction; UA, unstable angina.

Figure S 5: Patient pathways by cohort and index test (Cohorts 6 & 7)


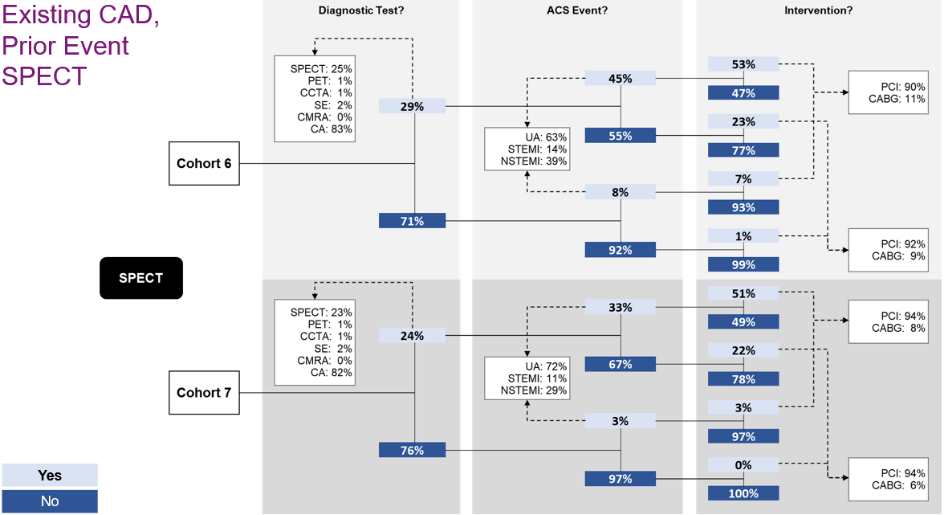

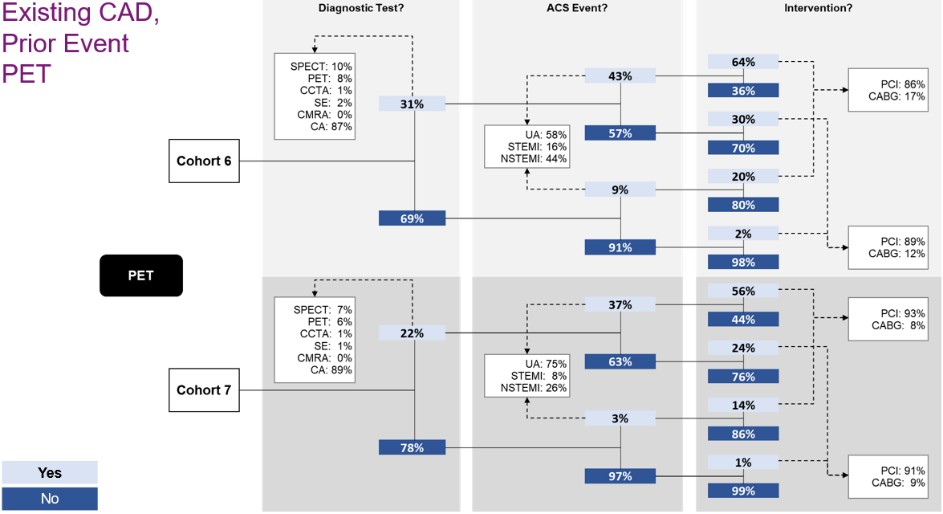


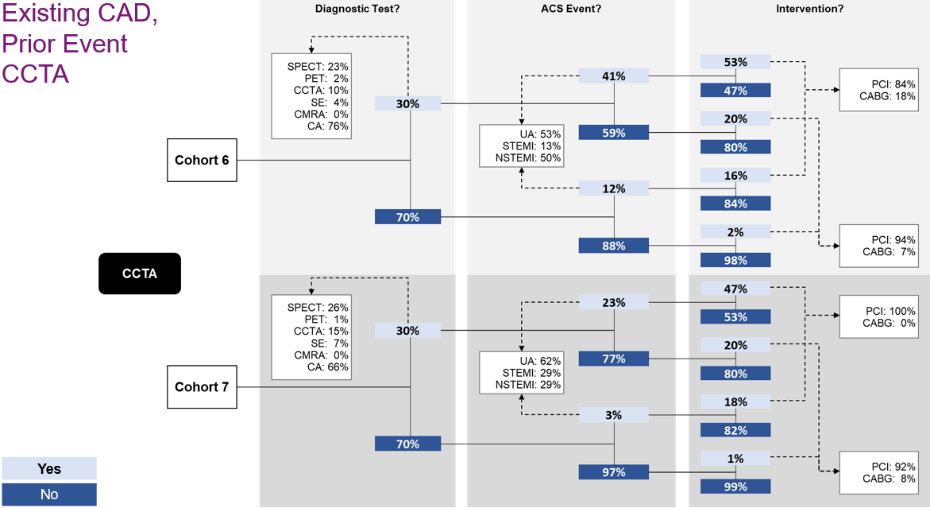

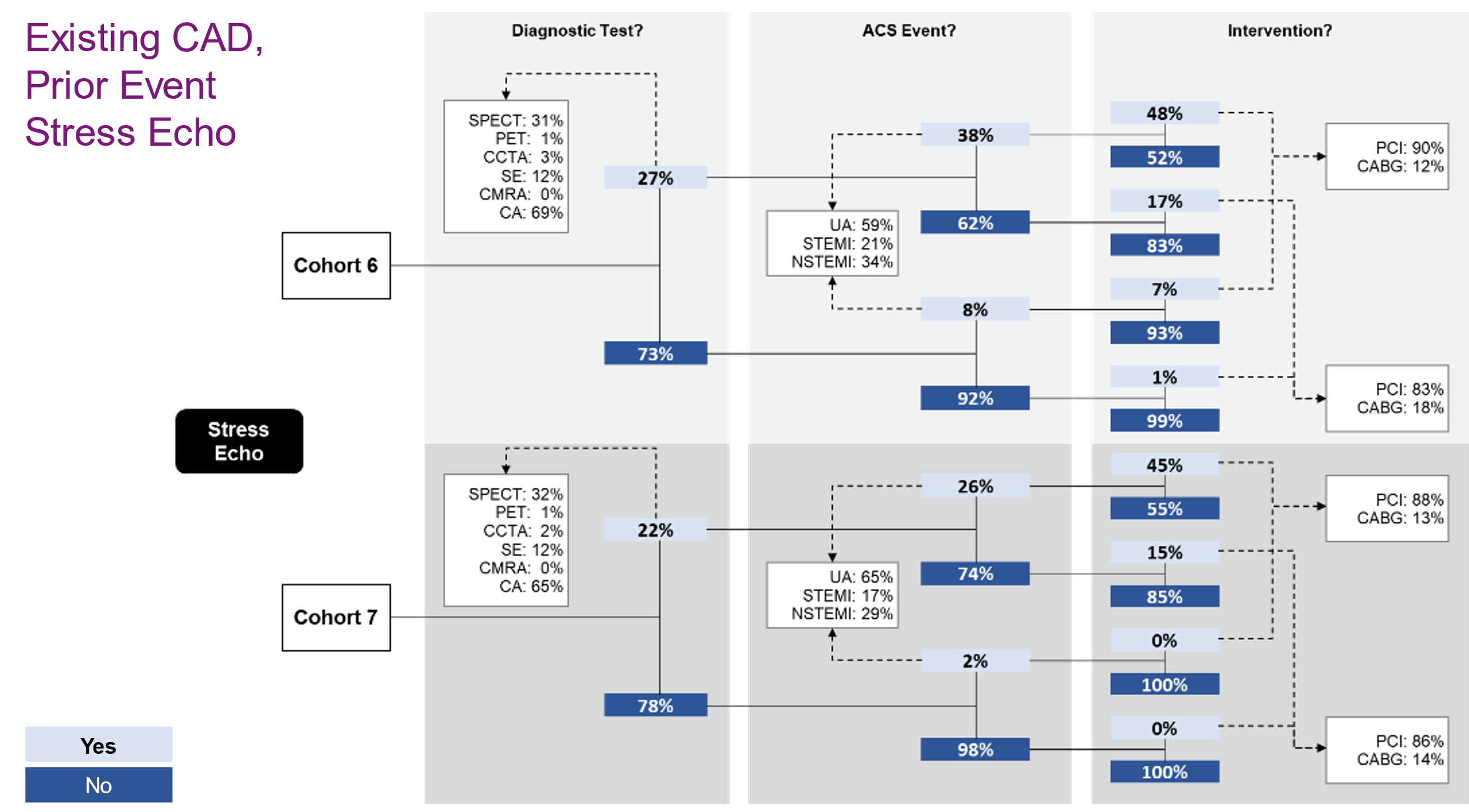


Abbreviations: ACS, acute coronary syndrome; CA, invasive coronary angiography; CABG, coronary artery bypass graft; CCTA, coronary computed tomography angiography; CMRA, coronary MR angiography; NSTEMI, non-ST-elevation myocardial infarction; PCI, percutaneous coronary intervention; PET, positron emission tomography; SE, stress echocardiography; SPECT, single-photon emission computed tomography; STEMI, ST-elevation myocardial infarction; UA, unstable angina.

Figure S 6: Patient pathways by cohort and index test (Cohorts 8 & 9)


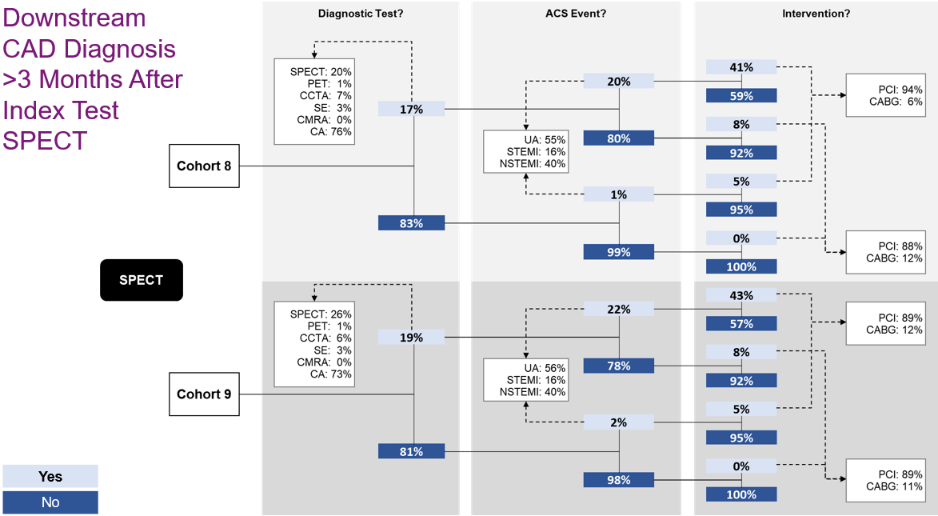

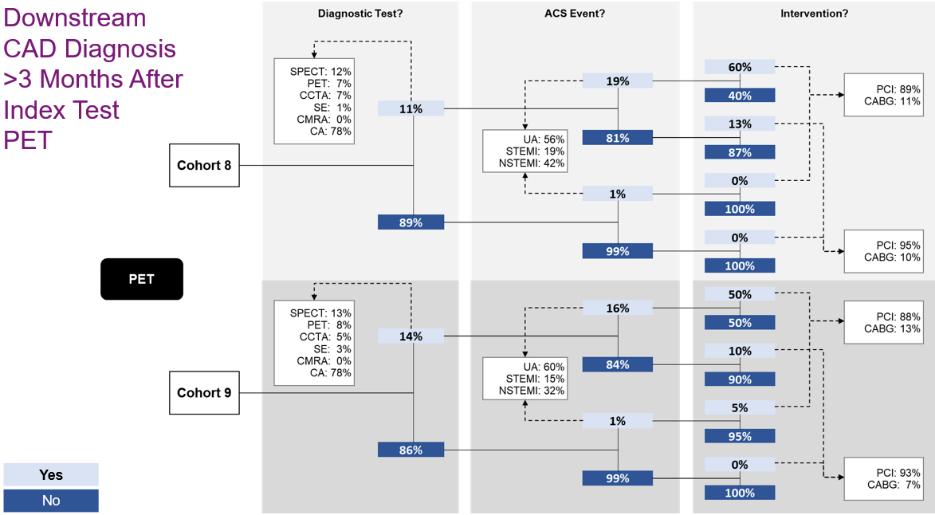


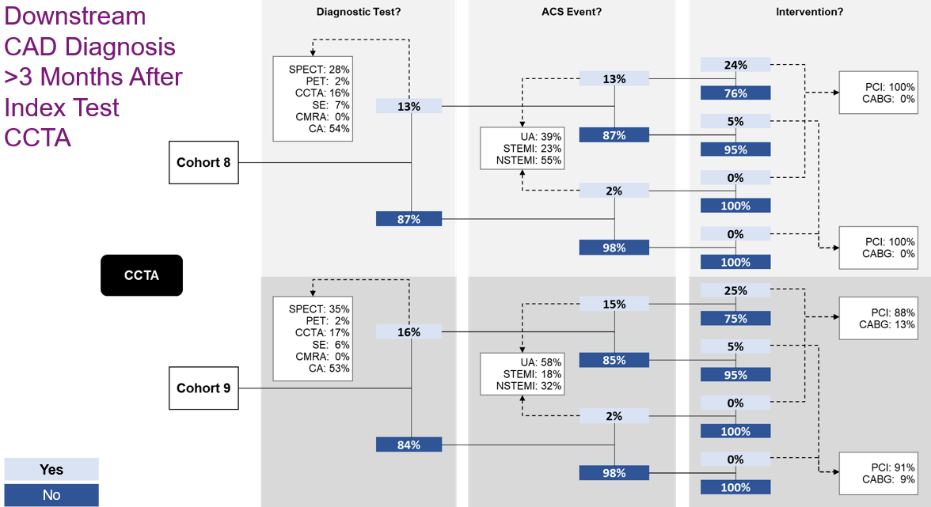

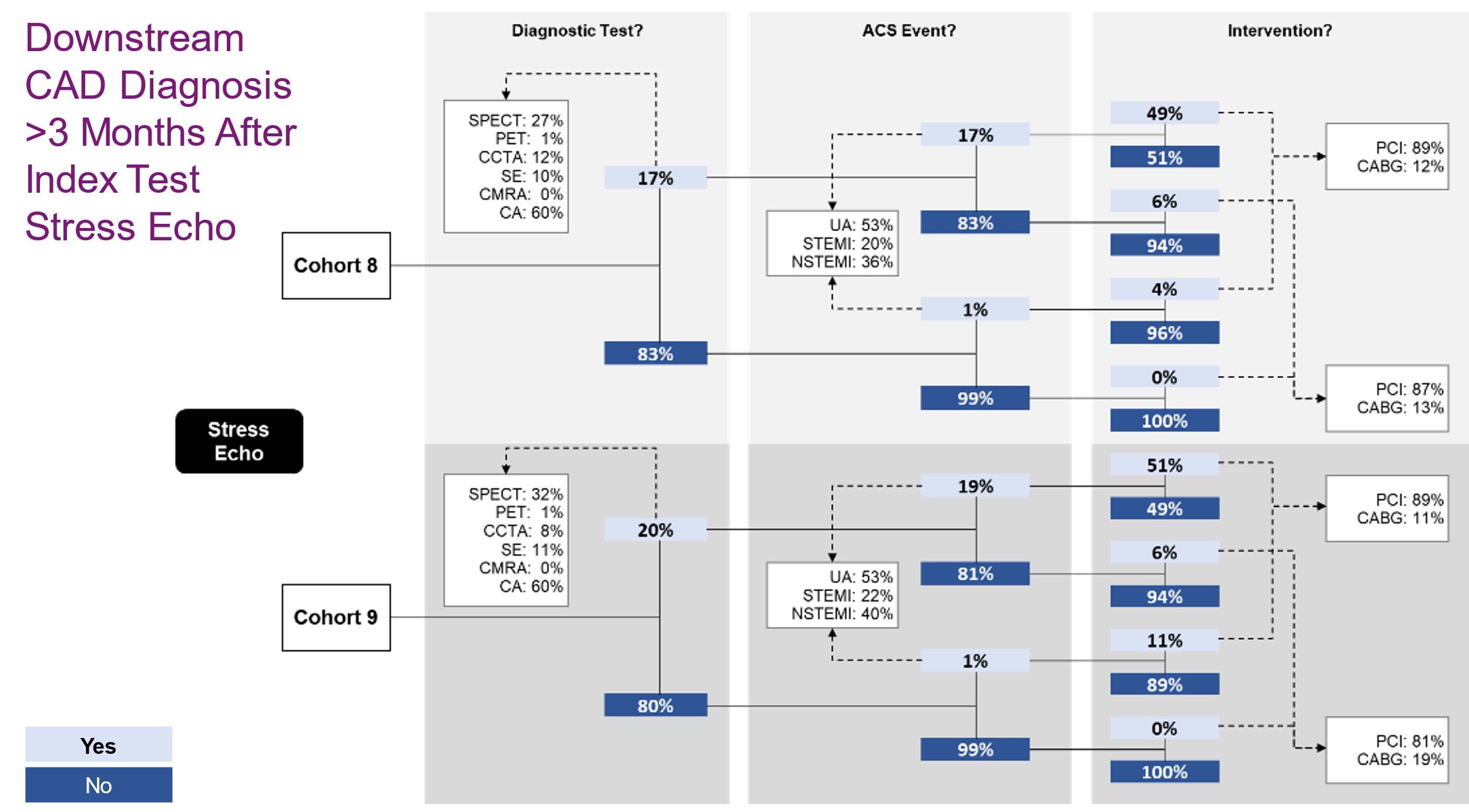


Abbreviations: ACS, acute coronary syndrome; CA, invasive coronary angiography; CABG, coronary artery bypass graft; CCTA, coronary computed tomography angiography; CMRA, coronary MR angiography; NSTEMI, non-ST-elevation myocardial infarction; PCI, percutaneous coronary intervention; PET, positron emission tomography; SE, stress echocardiography; SPECT, single-photon emission computed tomography; STEMI, ST-elevation myocardial infarction; UA, unstable angina.

Figure S 7: Proportions of patients with any downstream healthcare utilisation in those who underwent combinations of imaging tests


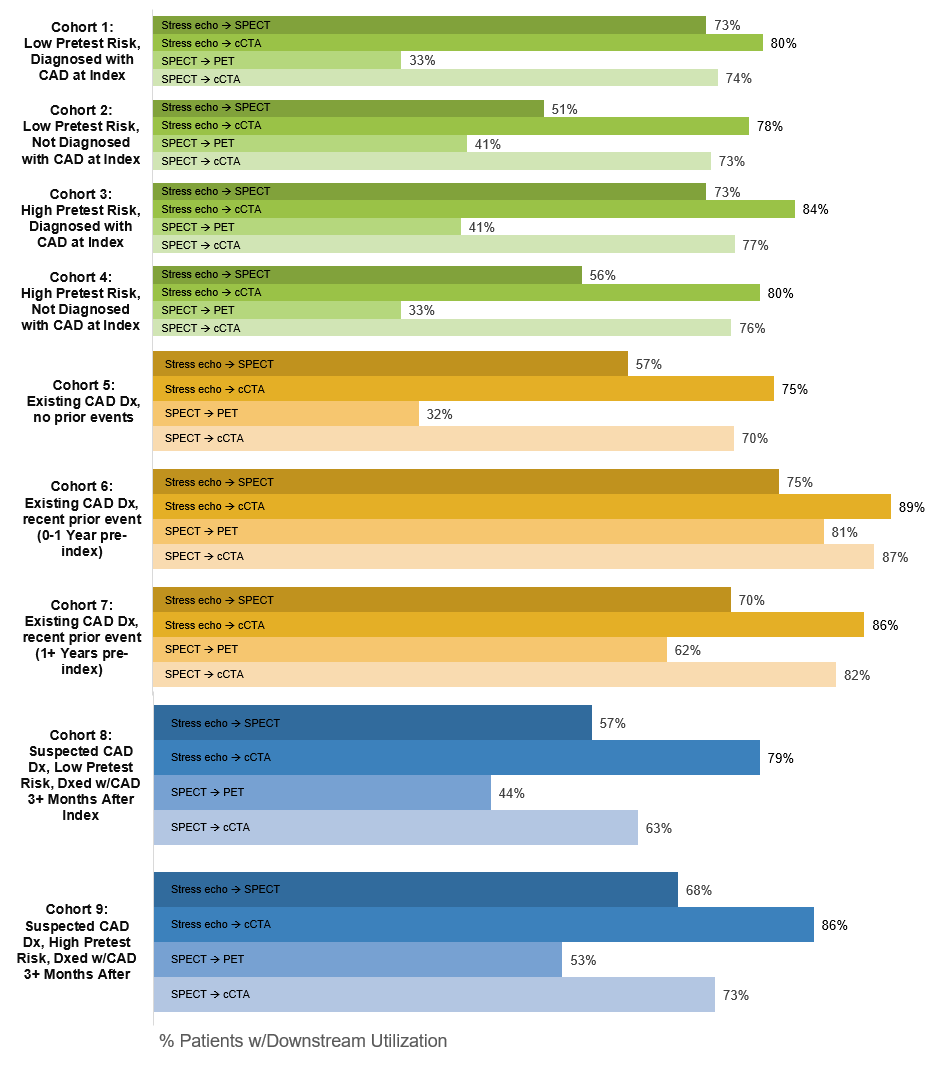


Abbreviations: CAD, coronary artery disease; cCTA, coronary computed tomography angiography; Dx, diagnosis; PET, positron emission tomography; SPECT, single-photon emission computed tomography.
